# Programming a Ferroptosis‐to‐Apoptosis Transition Landscape Revealed Ferroptosis Biomarkers and Repressors for Cancer Therapy

**DOI:** 10.1002/advs.202307263

**Published:** 2024-03-05

**Authors:** Yaron Vinik, Avi Maimon, Vinay Dubey, Harsha Raj, Ifat Abramovitch, Sergey Malitsky, Maxim Itkin, Avi Ma'ayan, Frank Westermann, Eyal Gottlieb, Eytan Ruppin, Sima Lev

**Affiliations:** ^1^ Molecular Cell Biology Department Weizmann Institute of Science Rehovot 76100 Israel; ^2^ The Ruth and Bruce Rappaport Faculty of Medicine Technion–Israel Institute of Technology Haifa 3525433 Israel; ^3^ Metabolic Profiling Unit Weizmann Institute of Science Rehovot 76100 Israel; ^4^ Department of Pharmacological Sciences Mount Sinai Center for Bioinformatics Icahn School of Medicine at Mount Sinai New York NY 10029 USA; ^5^ Neuroblastoma Genomics German Cancer Research Center (DKFZ) 69120 Heidelberg Germany; ^6^ Cancer Data Science Laboratory National Cancer Institute National Institutes of Health Bethesda MD 20892 USA

**Keywords:** apoptosis, biomarkers, breast cancer, classification signature, cancer therapy, ferroptosis, TNBC

## Abstract

Ferroptosis and apoptosis are key cell‐death pathways implicated in several human diseases including cancer. Ferroptosis is driven by iron‐dependent lipid peroxidation and currently has no characteristic biomarkers or gene signatures. Here a continuous phenotypic gradient between ferroptosis and apoptosis coupled to transcriptomic and metabolomic landscapes is established. The gradual ferroptosis‐to‐apoptosis transcriptomic landscape is used to generate a unique, unbiased transcriptomic predictor, the Gradient Gene Set (GGS), which classified ferroptosis and apoptosis with high accuracy. Further GGS optimization using multiple ferroptotic and apoptotic datasets revealed highly specific ferroptosis biomarkers, which are robustly validated in vitro and in vivo. A subset of the GGS is associated with poor prognosis in breast cancer patients and PDXs and contains different ferroptosis repressors. Depletion of one representative, PDGFA‐assaociated protein 1(PDAP1), is found to suppress basal‐like breast tumor growth in a mouse model. Omics and mechanistic studies revealed that ferroptosis is associated with enhanced lysosomal function, glutaminolysis, and the tricarboxylic acid (TCA) cycle, while its transition into apoptosis is attributed to enhanced endoplasmic reticulum(ER)‐stress and phosphatidylethanolamine (PE)‐to‐phosphatidylcholine (PC) metabolic shift. Collectively, this study highlights molecular mechanisms underlying ferroptosis execution, identified a highly predictive ferroptosis gene signature with prognostic value, ferroptosis versus apoptosis biomarkers, and ferroptosis repressors for breast cancer therapy.

## Graphical abstract

Transcriptomic, metabolomics, mechanistic, and computational analyses of programmed ferroptosis‐to‐apoptosis transition landscape identified ferroptosis selective biomarkers, revealed ferroptosis repressors as potential targets for breast cancer therapy, and highlighted ferroptosis‐toapoptosis molecular switches, including the PE/PC lipidomic shift.

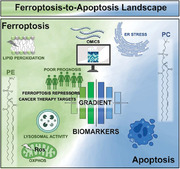

## 1 Introduction

Regulated cell death (RCD) pathways can be classified into apoptotic and non‐apoptotic death, such as necroptosis, autophagy, pyroptosis, and ferroptosis.^[^ ^1^ ^]^ Although different RCDs are mediated by distinct molecular mechanisms and characterized by unique morphological changes, increasing evidence suggests that RCDs can be connected through crosstalk and integrated signaling networks.^[^ ^2^ ^]^ Furthermore, different RCDs can be triggered by similar stress inducers, such as oxidative or ER stress, and recent studies suggest that dying cells may display molecular characteristics of more than one type of RCD, implying that “pure” selective or “mixed” RCD may exist.^[^ ^3^ ^]^ These observations highlight the need for a selective response signature for each RCD and specific biomarkers that can distinguish between different death modules, such as ferroptosis and apoptosis.^[^ ^4^ ^]^

Apoptosis, the most extensively studied RCD, has long been considered a promising target for cancer therapy, particularly since the discovery of B‐cell lymphoma 2 (BCL2) as an anti‐apoptotic oncogene.^[^ ^5^ ^]^ However, many cancer cells can escape apoptotic drugs and develop drug resistance.^[^ ^6^ ^]^ Increasing evidence suggests that ferroptosis could be a powerful therapeutic strategy for certain human cancers,^[^ ^7^ ^]^ and we and others have shown that among the different breast cancer subtypes, triple‐negative breast cancer (TNBC) is particularly susceptible to ferroptosis,^[^ ^8^ ^]^ and tumors from TNBC patients are enriched in ferroptosis‐related genes.^[^ ^8a^ ^]^

Ferroptosis is a unique RCD pathway driven by iron‐dependent lipid peroxidation and severe damage to cellular membranes.^[^ ^9^ ^]^ Labile iron (Fe2+) pool (LIP), polyunsaturated fatty acids (PUFAs), and reactive oxygen species (ROS) are fundamental for lipid peroxidation and ferroptosis execution.^[^ ^10^ ^]^ Unlike apoptosis, which is characterized by cell shrinkage, chromatin condensation, and membrane blebbing,^[^ ^1^ ^]^ ferroptotic cells mainly exhibit morphological changes in the mitochondria.^[^ ^11^ ^]^

Ferroptosis can be triggered by different mechanisms that modulate intracellular glutathione (GSH) pool, labile iron levels, PUFAs, and the activity of glutathione peroxidase 4 (GPX4), a selenoprotein that reduces phospholipid hydroperoxides (PLOOH) by glutathione.^[^ ^12^ ^]^ Since the initial discovery of erastin as an inhibitor of the cystine/glutamate antiporter (System x_c_^−^) and a ferroptosis inducer,^[^ ^9^ ^]^ many different ferroptosis inducers (FINs) and inhibitors were identified.^[^ ^13^ ^]^ Canonical FINs trigger ferroptosis through different mechanisms, including GSH depletion (erastin, artesunate), GPX4 inactivation (RSL3, ML210, ML162), or coenzyme Q10 (CoQ10) depletion (FIN56, CIL56).^[^ ^14^ ^]^ However, most of these FINs are effective in vitro but have low efficacy and/or high toxicity in vivo.^[^ ^15^ ^]^ We have recently identified a synthetic lethal drug combination targeting bromodomain‐containing protein 4  (BRD4) and the proteasome, which could also trigger ferroptosis in multiple TNBC cell lines and effectively inhibit tumor growth in xenograft and allograft mice models of TNBC.^[^ ^8a^ ^]^

In this study, we exploited the unique synthetic lethal drug combination targeting BRD4 (by JQ1) and the proteasome (by bortezomib) to induce ferroptosis in TNBC, and concurrently used a drug dose titration to program a continuous phenotypic gradient between ferroptosis and apoptosis. We showed that very low doses of JQ1 and bortezomib (BTZ) induced ferroptosis in basal‐like breast cancer through integrated responses mediated by enhanced lysosomal function, increased ferritinophagy, glutaminolysis, TCA cycle, oxidative phosphorylation, and activation of activating transcription factor 4 (ATF4), a master regulator of the integrated stress response (IRS) and amino acid metabolism.^[^ ^16^ ^]^ Ferroptosis‐to‐apoptosis transition was coupled to enhanced ER stress, XBP1, and ATF4/CHOP activation concomitant with a PE/PC‐ metabolic shift. Analysis of the gradual ferroptosis‐apoptosis transcriptomic landscape together with machine learning, public datasets of ferroptosis and apoptotic responses, as well as transcriptomic data from cancer patients and Patient‐derived xenografts (PDXs), led us to identify a unique Gradient Gene Set (GGS), which classified ferroptosis from apoptosis with high accuracy and could stratify breast cancer patients with poor prognosis. We also identified and validated a set of 24 selective ferroptosis versus apoptosis biomarkers and showed that depletion of PDAP1, a representative gene of the GGS, induced ferroptotic cell death and inhibited tumor growth in the xenograft model of basal‐like breast cancer.

## 2 Results

### 2.1 Programming a Ferroptosis to Apoptosis Transition Landscape

**Figure 1:**
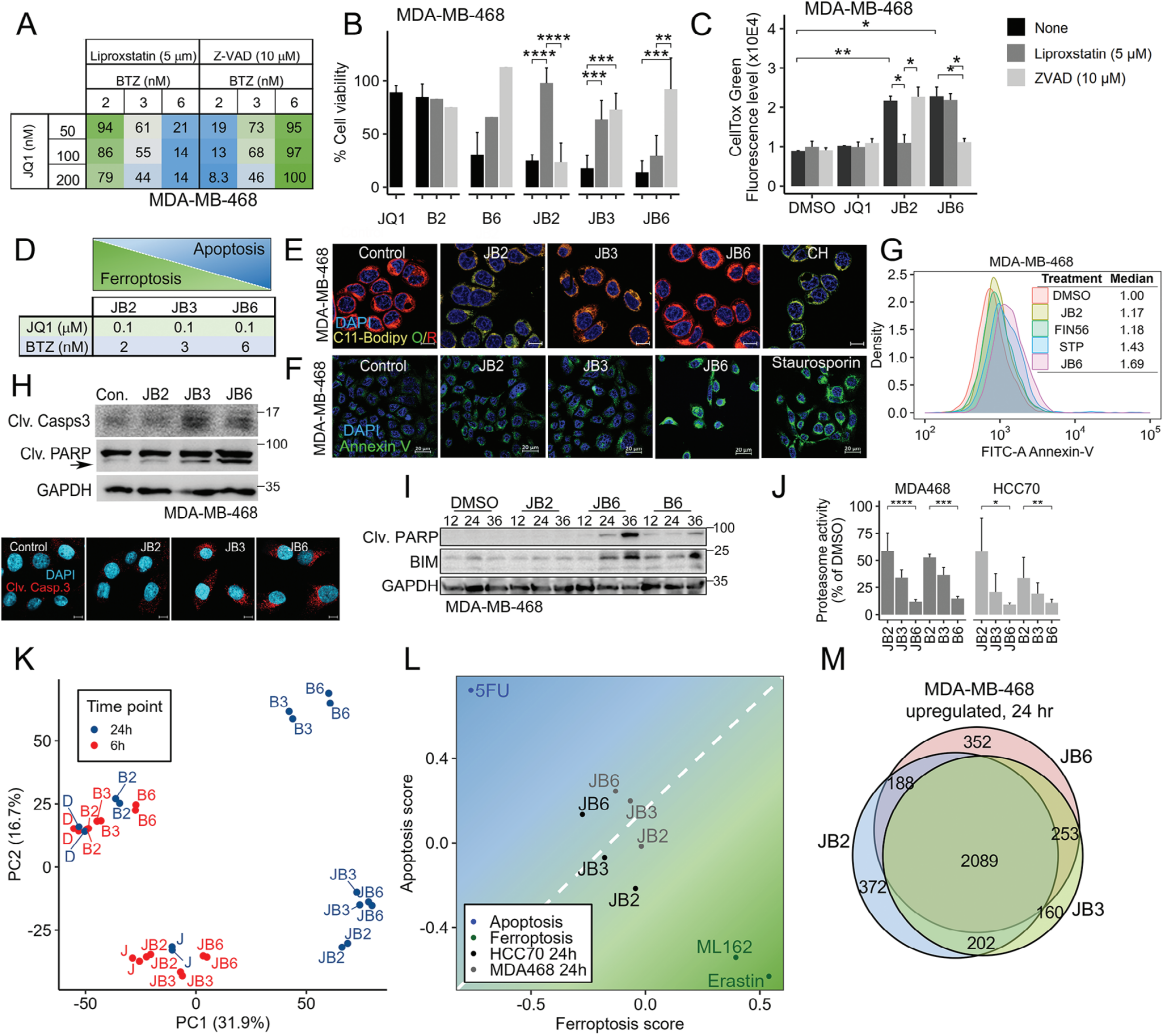
Programming of ferroptosis‐to‐apoptosis transition by JQ1 and bortezomib combination. (A) MDA‐MB‐468 basal‐like breast cancer cells were treated with the indicated concentration of JQ1 and bortezomib (BTZ) in the absence or presence of liproxstatin‐1 (5 µM) or z‐VAD‐FMK (10 µM) and cell viability was measured 72 h later by MTT. Percentageage (%) of cell viability was calculated compared to the control. (B,C) For JB combinations, JQ1 was applied at 100 nM while BTZ was at either 2 nM (JB2), 3 nM (JB3), or 6 nM (JB6). Cell viability (B) was measured by MTT, and cell death C) was measured by CellTox green. Shown are the mean values ± SD from at least 2 independent experiments (except for singles rescue). P‐values were measured by t‐test. (D) JB2, JB3, and JB6 are defined as the combinations of 100 nM JQ1 with 2 nM, 3 nM, and 6 nM BTZ, respectively. (E,F) Representative confocal images of MDA‐MB‐468 cells treated with the JB drug combinations and stained with C11‐BODIPY (E) or FITC‐Annexin V (F) Cumene hydroperoxide (CH; 75 µM) (D) or staurosporine (25 nM) (F) were used as positive controls. Scale bar, 10 µm (in E), 20 µm (in F). (G) FACS analysis of FITC‐Annexin‐V labeled MDA‐MB‐468 cells 24 h post‐treatment with the indicated drugs. (H–I) Cleavage of caspase‐3 (H), cleaved PARP, and BIM level (I) in response to drug treatment for 24 h (H) or for the indicated time points (in hours) (I) in MDA‐MB‐468 were assessed by Western Blotting (WB). Representative confocal images of cleaved caspase‐3 are shown in H (bottom). Scale bar, 10 µm. (J) Proteasome activity was assessed in MDA‐MB‐468 and HCC70 cells in response to the indicated drug treatment for 24 h. Cell lysates were subjected to proteasome activity using LLVY peptide reporter assay. Results are presented as % activity relative to control (DMSO). Shown are the mean values ± SD from 2 (MDA‐MB‐468) and 3 (HCC70) independent experiments done in duplicates. P‐values were measured by t‐test. (K–M) RNAseq was performed for the combinations JB2, JB3, and JB6, for single drug treatments (B2, B3, B6, J) and for DMSO (`D`) as control. (K) Principal component analysis of all treatments in the RNAseq was performed. Shown are the results for MDA‐MB‐468 cells. (L) All genes in each treatment were ranked based on the t‐statistics calculated for the fold change of gene expression in the treatment versus DMSO. GSVA was used to calculate the enrichment of a ferroptosis gene signature (x‐axis) and apoptosis gene signature (y‐axis) in JB2, JB3, and JB6 based on the t‐statistics ranks. The ferroptosis and apoptosis signatures were built for TNBC based on public datasets, using 5FU as a representative of apoptosis inducer, and erastin and ML162 as ferroptosis inducers. (M) Venn diagram of significantly upregulated genes (FDR < 0.05) in MDA‐MB‐468 cells in response to JB2, JB3, and JB6 treatments versus DMSO. *p‐value < 0.05, **p‐value < 0.01, ***p‐value < 0.001, ****p‐value < 1 × 10^−4^.

We previously showed that very low doses of JQ1 and BTZ induced ferroptosis across multiple TNBC cell lines.^[^ ^8a^ ^]^ To further assess the effects of JQ1 and BTZ on ferroptotic cell death, we slightly increased the levels of JQ1 (50–200 nM) and BTZ (2–6 nM), and examined cell viability (**Figure** **1**A,B; Figure S1A,B,D, Supporting Information) and cell death (Figure 1C; Figure S1C, Suppoting Information), in the absence or presence of either ferroptosis inhibitors (liproxstatin‐1, ferrostatin‐1), an apoptotic inhibitor (Z‐VAD) or a necrosis inhibitor (Necrostatin‐1) in two basal‐like breast cancer cell lines; MDA‐MB‐468 (Figure 1A–C; Figure S1D, Supporting Information) and HCC70 (Figure S1A–C, Supporting Information). As shown, the effect of JQ1 (50–200 nM) together with 2 nM BTZ was predominantly rescued by the ferroptosis inhibitors, while JQ1 (50–200 nM) with 3 nM BTZ was partially rescued by both liproxstatin‐1 and Z‐VAD, and JQ1 (50–200 nM) with 6 nM BTZ was mainly rescued by Z‐VAD. These results indicate that a minor gradual increase of BTZ from 2‐to‐3‐to‐6 nM could program a transition from ferroptosis to apoptosis. Accordingly, we proceeded with combinations of fixed, low JQ1 dose (100 nM) and increased doses of BTZ from 2 nM (JB2) to induce ferroptosis, 3 nM BTZ (JB3) to induce an intermediate “ferroapoptosis” state, and 6 nM (JB6) to induce apoptosis (Figure 1D). By using death pathway characteristic assays (Experimental Section),^[^ ^17^ ^]^ we showed that JB2 strongly induced lipid peroxidation, a hallmark of ferroptosis^[^ ^18^ ^]^ (Figure 1E; Figure S1E, Supporting Information), JB6 induced strong Annexin V staining (Figure 1F,G, Figure S1G, Supporting Information), upregulation of BIM level (Figure 1I) and cleavage of Caspase 3 and PARP (Figure 1H,I, Figure S1H, Supporting Information), all are hallmarks of apoptosis, while JB3 exhibit an intermediate phenotype. We also observed reduced levels of GPX4 and FSP1 proteins in JB2 (Figure S1I,Supporing Information), and a gradual inhibition in the proteasomal activity from JB2‐to‐JB6 (Figure 1J). Notably, Necrostatin‐1 could not rescue the effects of either JB6 or JB2 on cell viability (Figure S1D, Supporting Information), consistent with our previous report on JB2 and its specific effects on ferroptotic death across multiple TNBC cell lines, but not on normal‐like mammary cells (MCF10A) or luminal breast cancer cell line (MCF7).^[^ ^8a^ ^]^ Interestingly, JB6 also had a weaker effect in MCF7 compared to basal‐like cells (Figure S1D, Supporting Information) and didn't influence the sensitivity to ferroptosis (Figure S1J, Supporting Information).

We next coupled the gradual phenotypic transition of ferroptosis‐to‐apoptosis (JB2‐JB3‐JB6) to transcriptomic profiling. We performed RNAseq for MDA‐MB‐468 and HCC70 cells treated with individual drugs (100 nM JQ1 or 2, 3 or 6 nM BTZ; B2, B3, B6, respectively) and drug combinations (JB2, JB3, JB6) for 6 and 24 h (before cell death onset, Experimental Section). To visualize the transcriptomic changes induced by the different drug treatments, we performed principal component analysis (PCA) (Figure 1K, Figure S1K, Supporting Information). While JB2, JB3, and JB6 clustered together, they were distant from the control (DMSO), suggesting a large transcriptomic change in all three. The JB2‐JB3‐JB6 transcriptomic transition was further visualized on a plot whose axes represent gene set enrichment scores of ferroptosis^[^ ^19^ ^]^ versus apoptosis^[^ ^20^ ^]^ responses in TNBC cell lines of publicly available data. Enrichment analysis of the RNAseq data was measured by Gene Set Variation Analysis (GSVA) (Figure 1L). As shown, the three JB combinations were found in close proximity, with a gradual transcriptomic transition from ferroptosis (JB2) through the interphase (JB3) and into apoptosis (JB6), consistent with the parallel phenotypic transition. Finally, analysis of significant differentially expressed genes (DEGs) compared to DMSO, demonstrates that JB2, JB3, and JB6 share the vast majority (≈80%) of significant DEGs (Figure 1M; Figure S1L, Supporting Information), which represent a general stress response to the applied drug combinations regardless of the specific cell death module.

### 2.2 Identification of a Ferroptosis Versus Apoptosis Gene Signature

The transcriptomic landscapes induced by JB2 and JB6 revealed that only ≈20% of the DEGs can be specifically attributed to ferroptosis or apoptosis (Figure 1M), suggesting that these DEGs may segregate the transcriptomic response of ‐FINs‐ from apoptosis inducers (AINs).

**Figure 2:**
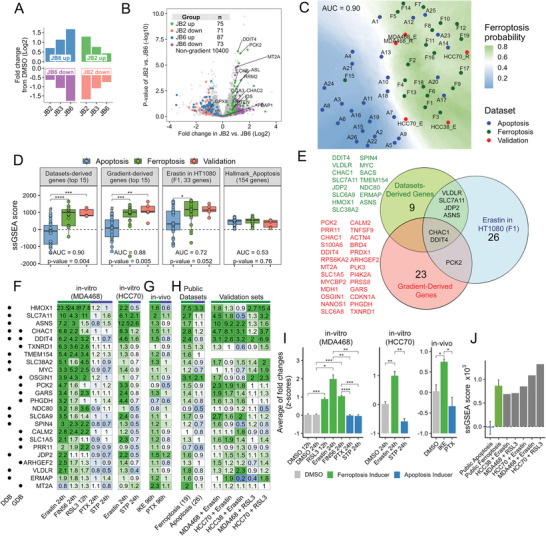
Establishment of ferroptosis versus apoptosis gene sets and biomarkers. (A) Average log2 fold change of JB2/3/6 versus DMSO for all the genes included in each of the GGS subsets. (B) Volcano plot highlighting the four subsets of the GGS. Data is based on RNAseq performed on MDA‐MB‐468 at 24 h. The insert table shows the number of genes in each subset (total = 306 genes). (C) UMAP projection of 19 ferroptosis inducers (green), 26 apoptosis inducers (blue), and 5 FIN validation sets (red) using the 306 genes of the gradient gene set (GGS). Shown is the AUC of classification between the ferroptosis and apoptosis inducers, calculated using k‐Nearest Neighbors. (D) Enrichment scores for the 15 top genes in the “Datasets‐derived” and the “Gradient‐derived” sets as well as the published response signature to erastin in HT1080 (*n* = 33 genes), and the hallmark apoptosis set from MsigDB (*n* = 154 genes). Scoring was determined by ssGSEA, in the public 19 ferroptosis and 26 apoptosis datasets (Table S1, Supporting Information), as well as in the validation sets of 5 FINs. ROC‐AUCs and *p*‐values of AUCs (at the bottom of each panel) were calculated for the FINs versus AINs classification. The Center line indicates the median of each group. `*` denotes p‐values measured by t‐test, between the FINs and AINs scores. `#` denotes non‐significant p‐values measured by a one‐sample t‐test, comparing the scores to zero (suggesting the genes are not significantly enriched in those sets). (E) Venn diagram showing the genes in the different gene sets. (F‐G) Shown are the selected 24 genes from the Datasets‐derived (DDB) and the Gradient‐derived (GDB) biomarkers that were used for validation by qRT‐PCR. For in‐vitro validation (F), MDA‐MB‐468 and HCC70 were treated with the indicated FINs (Erastin, FIN56, RSL3) and AINs (Paxlitaxel, Staurosporine), for the indicated time using IC_50_ concentrations. For in‐vivo validation (G), mice harboring MDA‐MB‐468 xenograft tumors were treated with IKE and paclitaxel as described in the experimental section. RNA was extracted and gene expression was examined by qPCR. Numbers are mean fold‐changes in gene expression versus DMSO of three independent repeats (in F), or three tumors per group (in G). Actin was used as a housekeeping gene for normalization. The genes are sorted by average fold changes in all FIN treatments. Points on the left mark the source of the biomarkers; Dataset‐derived (DDB) or Gradient‐derived (GDB) sets. (H) Expression of the 24 selected biomarkers in the RNAseq datasets used in this study. Left: the mean fold‐changes in the 19 FINs and 26 AINs public datasets. Right: Fold change in the 5 FIN validation datasets (averages of two repeats per set). (I) Integrated qRT‐PCR results of F‐G were performed by converting the fold changes values (vs DMSO) of the 24 genes into z‐scores (to normalize all the genes to the same scale), and then average the z‐scores of all 24 genes. Z‐scores were calculated by centering the fold‐changes around 1, with a standard deviation of 1. Shown are the means ± SD of three repeats or mice. *p*‐values determined by t‐test. (J) ssGSEA enrichment scores for the 24 biomarkers in the public datasets (averages) and the 5 FIN validation sets.

To explore this possibility, we used the RNAseq data of JB2 (ferroptosis) and JB6 (apoptosis) at 24 h to generate four gene sets, each containing genes that were significantly up or down‐regulated in either JB2 or JB6 but not in both. For example, the “JB2 Up” set included genes that were significantly upregulated (FDR < 0.05) in JB2 versus DMSO but not significant in JB6 versus DMSO. This gene set was then filtered to include only the genes with the highest significant difference (FDR < 0.05) between JB2 and JB6 (**Figure** **2**A,B, Figure S2A, Supporting Information). Notably, this gene set included genes associated with GSH metabolism, TCA cycle, and lysosome (Figure S2B, Supporting Information), among others.

We then combined the four gene sets (JB2 Up/Down; 146 genes, JB6 Up/Down; 160 genes, a total 306 genes) to form the “Gradient Gene Set” (GGS), a unique gene set of the most differential transcriptomic response between JB2 (ferroptosis) and JB6 (apoptosis) (Figure 2B, Figure S2A, Supporting Information). We hypothesized that the GGS can classify the transcriptomic response of ferroptosis distinctly from apoptosis. To test this hypothesis, we collected transcriptomic data from 19 FINs and 26 AINs‐from the GEO datasets (Table S1, Supporting Information). In addition, we performed RNAseq analysis of classical FINs (erastin and RSL3) responses in basal‐like breast cancer cell lines (MDA‐MB‐468, HCC70, and HCC38), and combined these five datasets (validation group) to the 45 (19 FINs, 26 AINs) public datasets. UMAP was used to visualize the expression of the 306 GGS genes in these 50 datasets (Figure 2C). This analysis generated a map with distinct ferroptosis and apoptosis regions and a clear decision boundary in between. Importantly, the five validation datasets of FINs responses were mapped in the ferroptosis area (Figure 2C, red dots). Furthermore, a K‐nearest neighbors analysis showed the high classification power of the GGS, which accurately classified the ferroptosis and apoptosis datasets (ROC‐AUC = 0.90) (Figure 2C).

### 2.3 Optimization of Ferroptosis Versus Apoptosis Biomarkers

Although the GGS could classify ferroptosis from apoptosis with high accuracy, it consists of a relatively high number of genes and thus can be useful to classify RNAseq data. To define a smaller subset of genes that can be used as ferroptosis versus apoptosis biomarkers, we selected various gene sets of different sizes according to their differential expression (fold change) between JB2 (ferroptosis) and JB6 (apoptosis) at two‐time points (6 h, 24 h). We then measured the enrichment of each gene set in the 19 FINs and 26 AINs datasets by ssGSEA and scored their classification accuracy by ROC‐AUC.

We found that the best AUC is given by a set of genes whose transcriptomic gradient shifts from 6 to 24 h: their expression was significantly lower in JB2 versus JB6 at 6 h but significantly higher in JB2 versus JB6 at 24 h (Figure S2C, Table S2, Supporting Information). This set consists of 26 genes and was termed the “Gradient derived” genes set. We assume that the decreased expression at an early time point may induce the onset of ferroptosis, while the increase at a later time may represent an attempt to compensate and protect from ferroptosis. The “Gradient derived” genes set had a strong and significant classification accuracy between the 19 FINs and 26 AINs datasets (AUC = 0.88, Figure 2D; Figure S2E, Supporting Information). It was also highly enriched in the validation group of the 5 FINs response datasets of basal‐like TNBC cell lines (Figure 2D) and thus can be considered as a powerful ferroptosis to apoptosis classifier.

To further corroborate the “Gradient derived” classification capacity, we compared it to the best possible classifier between the 19 FINs and 26 AINs datasets. To this end, we generated a set of 15 genes, termed the “Datasets derived` genes set, by ranking all the genes in the datasets according to their relative expression in the FINs versus the AINs datasets, using a bootstrapped aggregation method (Figure S2D, Supporting Information). This analysis revealed that the classification accuracy of the top‐ranked 15 genes was very similar (AUC = 0.9) to the top 15 genes of the ‘Gradient derived” set (Figure 2D). Nevertheless, increasing the number of the “Datasets derived” genes above 15 (Table S2, Supporting Information), concurrently increased the classification accuracy (AUC) (Figure S2E, Supporting Information) and, thus could also be useful when full transcriptomic data is available.

Collectively, both the “Gradient derived” and the “Datasets derived” ferroptosis versus apoptosis biomarkers display high classification accuracy between the FINs and AINs datasets (Figure S2F, Supporting Information), and could accurately classify the JB2, JB3, and JB6 transcriptomic landscape (Figure S2G, Supporting Information). Furthermore, their enrichment scores in the AINs datasets were negligible (almost zero) (Figure 2D), highlighting their specificity to ferroptosis. Of note, a set of 33 genes (Table S2, Supporting Information), which was generated in response to erastin in HT‐1080 (“Erastin/HT1080 derived”),^[^ ^21^ ^]^ and is often considered a ferroptotic biomarkers set,^[^ ^22^ ^]^ was found to be highly enriched in the FINs datasets, as expected, but also in several AINs datasets, exhibiting a lower AUC between the FINs and AINs datasets (AUC = 0.72, Figure 2D, third panel). Similarly, the commonly used “hallmark_apoptosis”, a set of 154 genes (MsigDB), was enriched both in the AINs and the FINs datasets, suggesting a weak specificity (Figure 2D, fourth panel).

The high accuracy and specificity of the “Gradient derived” and the “Datasets derived” ferroptosis versus apoptosis biomarkers (Figure 2D), which share only two genes (CHAC1, DDIT4) (Figure 2E; Table S2, Supporting Information), highlight their strength as reliable biomarkers. For validation, we selected the 15 and 12 top‐ranked genes of the “Datasets derived” and “Gradient derived” gene sets and composed a set of 24 genes (2 of the 27 are common, and one gene, SACS, was removed due to high variation in the qRT‐PCR results) with the best ferroptosis versus apoptosis prediction. We then experimentally validated the accuracy of this ferroptosis‐optimized gene set by qPCR (Figure 2F,G). For in vitro validation studies, we used mRNA of MDA‐MB‐468 or HCC70 cells treated with either classical FINs; erastin, FIN56, and RSL3, or with known apoptosis inducers; paclitaxel and staurosporine, as indicated (Figure 2F; Figure S3A, Supporting Information). For the in vivo validation, we established orthotopic xenograft mouse models by implanting MDA‐MB‐468 in the mammary fat pad of nude mice. When tumors reached ≈50 mm^3^, the mice were treated with either imidazole ketone erastin (IKE) or with paclitaxel (PTX) as described in the experimental section, to induce ferroptosis or apoptosis, respectively (Figure S3B, Supporting Information), and the expression levels of the 24 biomarkers were assessed by qRT‐PCR (Figure 2G). We then calculated the average fold changes for each in vitro and in vivo treatment. As seen in Figure 2I, the expression of the 24 biomarkers was significantly and specifically increased in response to ferroptosis inducers but not in response to the apoptotic inducers both in vitro and in vivo (Figure 2F,G,I).

These results were further corroborated by the high expression levels and ssGSEA enrichment scores of the 24 biomarkers within the 19 FIN datasets and five validation RNAseq datasets of FINs (Figure 2H,J). Overall, our comprehensive validation demonstrates the power, specificity, and predictive value of this optimized list of 24 ferroptosis versus apoptosis biomarkers, which can be used as reliable, universal ferroptosis selective biomarkers and can be tested by several qPCRs.

Importantly, we propose to validate a few biomarkers instead of individual genes due to different modes of action of various chemical FINs, each displaying slightly different kinetics (Figure S3C, Supporting Information) and intensity (fold change) in their transcriptomic response of these biomarkers as well as different kinetics in lipid peroxidation (Figure S3D,E, Supporting Information). Furthermore, ferroptosis induction by a nonchemical genetic mode (shRNA, gene knockdown, siRNA), might induce lipid peroxidation at different time windows due to adaptation responses. This could influence their transcriptomic response and the enrichment of the 24 biomarkers as shown for the GPX4 knockdown dataset (Figure S2F, Supporting Information, point #F11).

### 2.4 A GGS Subset Exhibits a Prognostic Value

**Figure 3:**
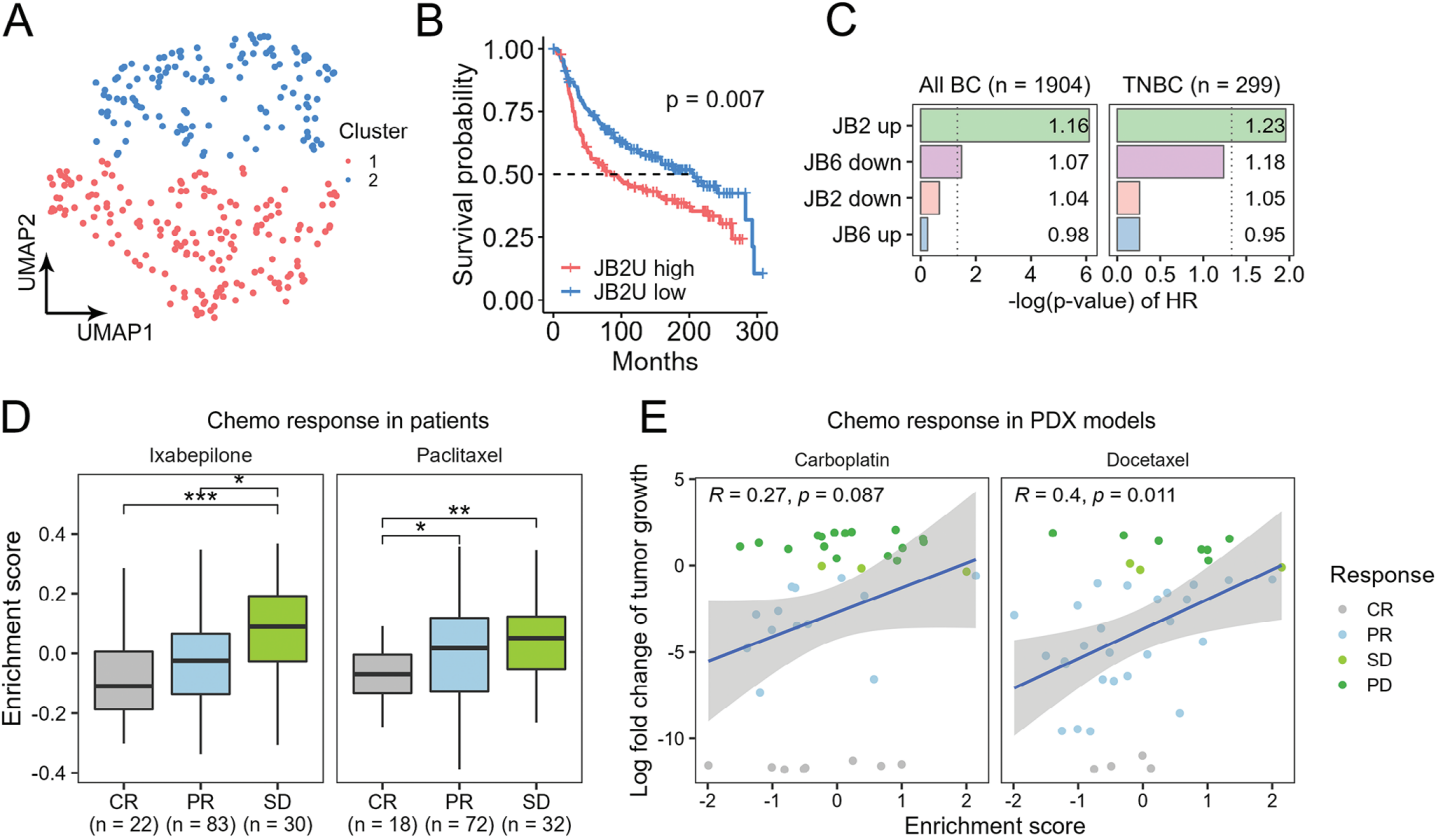
A GGS subset exhibits a prognostic value. A,B) Enrichment of the four GGS subsets (JB2/6 Up/Down) was scored in human TNBC patients (*n* = 299, METABRIC) by ssGSEA. The scores are visualized by UMAP (A). Kmeans clustering was used to cluster the patients into two clusters. Kaplan‐Meier analysis was used to show the survival probabilities of patients in both clusters (B). p‐value in B was measured by a log‐rank test. C) Hazard ratios (HR) were calculated for the GGS subsets in breast cancer patients (*n* = 1904 patients) and only in TNBC patients (*n* = 299 patients; METABRIC dataset). Quantification was done using Cox proportional hazards model. Signature scoring in each patient was done using ssGSEA. Bar graphs show the p‐values of the HR; the numbers in each bar are the HR values. (D) ssGSEA was used to quantify the enrichment of the 75 `JB2 Up` genes in a dataset containing *n* = 279 patients treated with ixabepilone or paclitaxel (data taken from GSE41998). Patients were grouped based on the therapy outcome: CR‐complete response; PR‐partial response; and SD‐stable disease. *p*‐values were determined by t‐test. (E) ssGSEA was used to quantify the enrichment of the 75 `JB2 Up` genes in a dataset containing 40 PDX models. PDX models were grouped based on the therapy outcome: CR‐complete response; PR‐partial response; SD‐stable disease, PD‐progressive disease. Pearson's correlations are shown with their p‐values. *p‐value < 0.05, **p‐value < 0.01, ***p‐value < 0.001.

We next examined the enrichment of the GGS signature (by ssGSEA) in TNBC patients from the METABRIC cohort (*n* = 299). UMAP analysis of the enrichment scores revealed two major clusters of patients (**Figure** **3**A), which displayed significant differences in the enrichment of the “JB2 Up” subset (Figure S4A, Supporting Information) and concomitantly in their survival as determined by Kaplan‐Meier analysis (Figure 3B). This was further corroborated by univariate (Figure 3C) and multivariate (Figure S4B, Supporting Information) Cox proportional hazard models, which revealed that the “JB2 Up” gene set is highly correlated with poor prognosis in breast cancer patients (TNBC and non‐TNBC). These findings suggest that tumors with high enrichment of the “JB2 Up” genes are more aggressive. Indeed, high enrichment of “JB2 Up” gene set was obtained in chemoresistant breast cancer patients (*n* = 257)^[^ ^23^ ^]^ (Figure 3D) and was further corroborated in 40 PDX models of breast cancer, with known transcriptomic data and response to chemotherapy.^[^ ^24^ ^]^ As shown, we observed a high correlation between the enrichment of “JB2 Up” and the response of the PDXs to docetaxel and carboplatin treatments (Figure 3E). Taken together, we identified a subset of GGS, the “JB2 Up” genes, as a potential prognostic gene set for breast cancer aggressiveness and prediction of chemotherapy response.

### 2.5 A GGS Subset Contains Potential Ferroptosis Repressors For Breast Cancer Therapy

**Figure 4:**
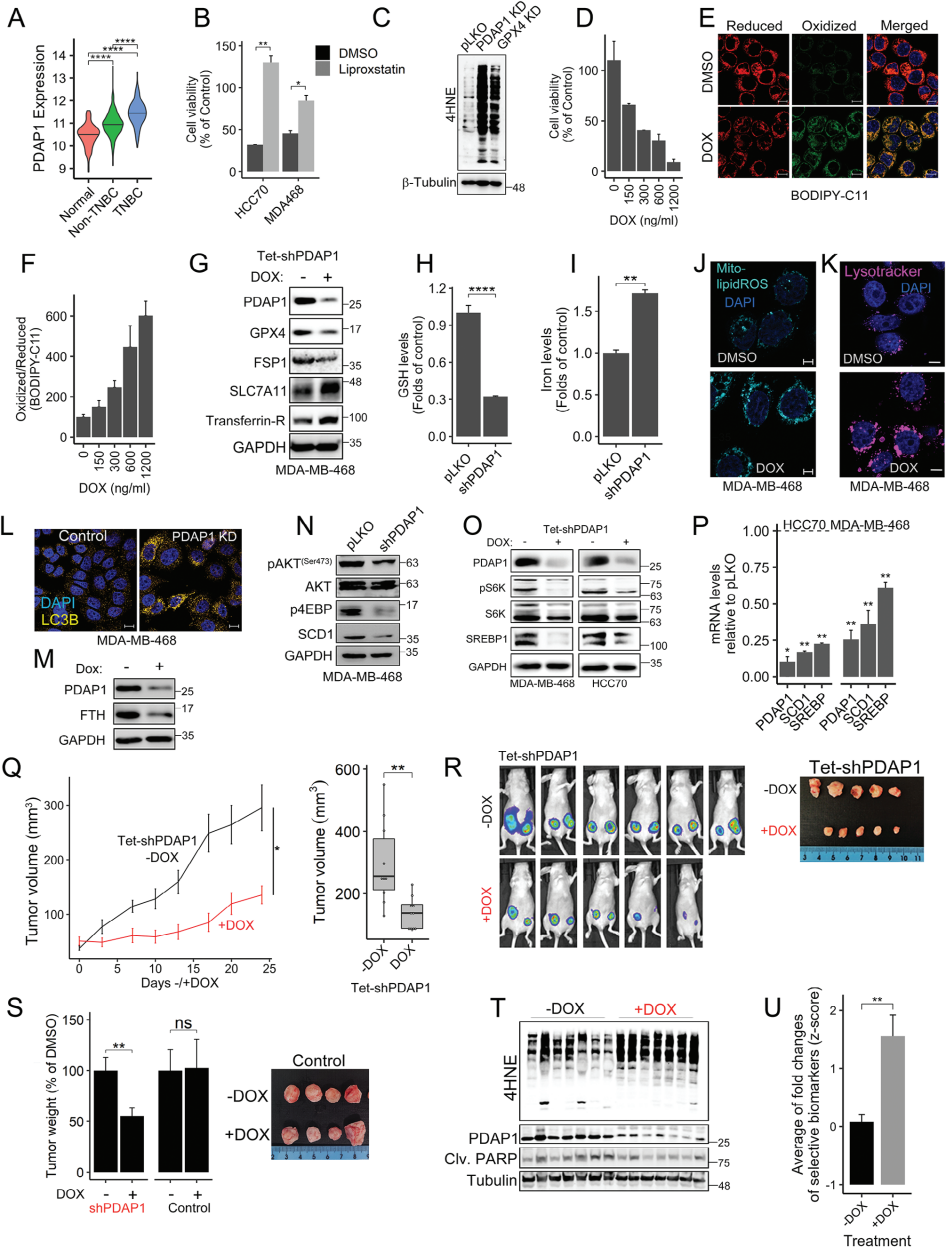
PDAP1 depletion induced ferroptosis and inhibited breast tumor growth in mice. (A) PDAP1 expression in breast cancer patients. Data was taken from TCGA (*n* = 114 normal, 879 non‐TNBC, 180 TNBC). *p*‐values were calculated by t‐test. (B) Knockdown of PDAP1 reduced cell viability. MDA‐MB‐468 and HCC70 cells depleted of PDAP1 (shPDAP1) were grown in the absence or presence of liproxstatin‐1 and cell viability was assessed 72 h later. Cell viability was calculated relative to control cells (pLKO) (%) and mean values ± SD from two independent experiments are shown. *p*‐values were measured by t‐test. (C) Control MDA‐MB‐468 cells (pLKO) and PDAP1 or GPX4 knockdown (KD) cells were analyzed for 4‐NHE‐protein adducts by WB with ß‐tubulin as a loading control. (D‐F) MDA‐MB‐468 cells expressing an inducible shPDAP1 (Tet‐pLKO‐shPDAP1) were treated with doxycycline (DOX) as indicated and assessed for either cell viability 96 h later (D) or for lipid‐ROS 48 h later (E, F). Cell viability and fluorometric quantification of lipid‐ROS were calculated relative to DOX untreated (DMSO) control and the mean values ± SD from two independent experiments are shown. Representative confocal images of BODIPY‐C11 fluorescence are shown (E). Scale bar, 10 µm. (G) Western blot showing the levels of ferroptosis‐related proteins in control and PDAP1 depleted cells (± DOX). (H) GSH levels were quantified in PDAP1 KD MDA‐MB‐468 cells. Shown are means (relative to pLKO) ± SD of 3 experiments done in duplicates. *p*‐values were measured by t‐test. (I) Intracellular iron levels were assessed by colorimetric assay in control and PDAP1‐KD. Shown are mean values ± SD of 2 independent repeats. p‐values were measured by t‐test. (J, K) Representative confocal images of control (DMSO) and PDAP1 depleted (DOX) MDA‐MB‐468 cells stained with the mitochondrial lipid peroxidation probe MitoPeDPP (J) or with lysotracker (K). Scale bar, 5 µm. (L) Control (pLKO) or PDAP1 KD MDA‐MB‐468 cells were analyzed for LC3B expression by immunofluorescence. Scale bars 10 µm. (M) Western blot analysis of FTH level in control (‐DOX) and PDAP1 depleted MDA‐MB‐468 cells (+ DOX). (N, O) Western blot analysis of the indicated signaling components in control (pLKO/‐DOX) and PDAP1 KD (shPDAP1/+DOX) MDA‐MB‐468 and/or HCC70 cells. (P) Transcripts of SREBP and SCD1 were assessed by qPCR. Mean values ± SD of two independent experiments are shown. p‐values were calculated by t‐test. (Q‐S) Depletion of PDAP1 reduced tumor burden and increased lipid‐ROS in vivo. Luciferase‐labeled MDA‐MB‐468 cells expressing the Tet‐pLKO‐shPDAP1 were implanted bilaterally into the mammary fat pad of female nude mice. When tumors reached ≈50 mm^3^, mice were randomized into two groups; shPDAP1 (+DOX) and non‐induced (‐DOX), *n* = 10 tumors/group (Q). Parallel experiment (S) was performed with scramble shRNA instead of PDAP1 shRNA (shScramble) as control (*n* = 6 tumors/group). (Q) The left graph shows the mean tumor volume ± SEM in the indicated time points from non‐induced (−DOX) and induced (+DOX) shPDAP1 tumors. Statistical significance was determined by 2‐way ANOVA (*p‐value < 0.05). The right Boxplot shows the tumor volumes at the experimental endpoint (24 days post‐induction with DOX). *p*‐values were measured by t‐test. (R) Representative bioluminescence imaging of xenograft tumors at day 24 after DOX induction (left), along with the excised tumors (right) showing the reduced size of PDAP1 KD tumors. (S) Weights of PDAP1 depleted (+DOX) or not‐depleted (‐DOX) tumors (day 24), and of Control (scrambled shRNA) tumors ± DOX. Shown are the mean mass of excised tumors ±SEM. T‐test was used to measure statistical significance. Right: Excised tumors from mice harboring the control (scrambled) shRNA treated with or without DOX. (T) Western blot analysis of tumors expressing the inducible shPDAP1 in the presence (+) or absence (−) of DOX (*n* = 7). Tumors were homogenized, lysed and analyzed for protein levels of PDAP1, 4HNE‐protein adducts, and cleaved PARP. β‐Tubulin was used as a loading control. (U) RNA was extracted from the tumors, and qRT‐PCR was used to quantify the expression levels of 21 out of the 24 ferroptosis versus apoptosis biomarkers (shown in Figure 2F). Plot shows the average z‐scores (fold changes centered ≈1) of the 21 genes (actual fold changes are shown in Figure S5J). t‐test was used to measure p‐value. *p‐value < 0.05, **p‐value < 0.01, ***p‐value < 0.001, ****p‐value < 1×10^−4^.

The finding that the “JB2 Up” set of 75 ferroptosis upregulated genes has prognostic value (Figure 3) suggests that it may contain ferroptosis repressors. Indeed, a few genes of this subset have already been shown to suppress ferroptosis, such as RRM2,^[^ ^25^ ^]^ DNM1^[^ ^26^ ^]^ and PCK2.^[^ ^27^ ^]^ As a proof of concept, we validated one of these genes, PDAP1 ‐. Previos studies showed that PDAP1 is overexpressed and/or involved in the progression of several cancers including gastric cancer, rectal carcinoma, glioma, and colorectal cancer.^[^ ^28^ ^]^ We selected PDAP1 since it is significantly highly expressed in breast cancer patients, particularly in TNBC (**Figure** **4**A). Furthermore, we found that its knockdown (KD) induced a very similar transcriptomic response of GPX4 KD based on the Connectivity Map (Broad,^[^ ^29^ ^]^ Figure S5A, Supporting Information), and thus, could be a promising candidate for further analysis.

Indeed, depletion of PDAP1 expression by shRNA in basal‐like TNBC cell lines MDA‐MB‐468 and HCC70 (Figure S5B, Supporting Information) markedly reduced cell survival, which could be rescued by liproxstatin‐1 (Figure 4B) or ferrostatin‐1 (Figure S5C, Supporting Information). PDAP1 depletion increased the levels of 4‐hydroxynonenal (4‐HNE) modified proteins, comparable to shGPX4 (Figure 4C) and of oxidized BODIPY‐C11 (Figure S5D, Supporting Information). Similar effects were observed with the doxycycline (DOX) inducible PDAP1 KD (Tet‐pLKO‐shPDAP1) (Figure 4D–F). The increased lipid peroxidation in PDAP1‐depleted cells was accompanied by reduced levels of GPX4 and FSP1 proteins (Figure 4G), reduced levels of intracellular GSH (Figure 4H), and elevated levels of iron (Figure 4I), TFRC (Figure 4G), and lipid ROS in the mitochondria (Figure 4J), which all can contribute to ferroptosis cell death.

We further found that exogenous GSH suppressed lipid peroxidation in PDAP1‐KD cells (Figure S5E, Supporting Information) and almost restored their cell viability (Figure S5F, Supporting Information), similar to the effect of the iron chelator 2,2′‐bipyridine (Figure S5G, Supporting Information). The elevated cellular iron levels in PDAP1‐depleted cells were associated with increased intensity of lysosomal staining (Figure 4K) and LC3B (Figure 4L), an autophagic activity marker, reduced level of ferritin (Figure 4M) and increased level of lysosomal iron, as shown by ferro‐orange staining (Figure S5H, Supporting Information). These results suggest that PDAP1 depletion enhanced ferretinophagy and consequently increased the labile iron pool.

It is well known that autophagy is negatively regulated by mTOR,^[^ ^30^ ^]^ and previous studies suggest that PDAP1 is an upstream regulator of AKT,^[^ ^31^ ^]^ implying that PDAP1 regulates the AKT‐mTOR pathway. Indeed, we found that PDAP1 KD reduced the phosphorylation of AKT1 (S473), of 4EBP and S6 kinase, two mTORC1 downstream targets (Figure 4N; Figure S5I, Supporting Information). We also observed a reduced level of stearoyl‐CoA desaturase‐1‐(SCD1) protein, the rate‐limiting enzyme of monounsaturated fatty acids (MUFAs) biosynthesis, and of SCD1 transcript (Figure 4N–P; Figure S5I, Supporting Information), which is regulated by the ‐Sterol regulatory element binding protein 1 (SREBP1) transcription factor, a downstream effector of AKT‐mTOR pathway.^[^ ^32^ ^]^ Importantly, both the protein and mRNA levels of SREBP1 were also reduced in PDAP1‐depleted MDA‐MB‐468 or HCC70 cells (Figure 4O, 4P). Collectively, these results suggest that PDAP1 can protect from ferroptotic death through the AKT‐mTOR‐SREBP1‐SCD1 signaling axis. Its depletion increased lysosomal activity, autophagy, and ferritinophagy, thereby elevating labile iron. Concurrently, PDAP1 depletion decreased SREB1 and SCD1 to possibly attenuate the mevalonate pathway and to elevate PUFAs. These effects together with reduced GSH levels, GPX4 and FSP1 can contribute to ferroptosis execution in PDAP1‐KD cells.

We then examined the impact of PDAP1 depletion on tumorigenesis in a xenograft model of MDA‐MB‐468 cells. MDA‐MB‐468 cells harboring the Tet‐pLKO‐shPDAP1 or shScrambled (control) were orthotopically implanted into the mammary fat pad of athymic nude mice (*n* = 10 tumors), and tumor growth was monitored over time by caliper as described in methods. When tumors reached ≈50 mm^3^ (≈3–4 weeks post‐implantation), the mice were randomized into control and doxycycline (DOX) untreated and treated groups to induce PDAP1 KD. As shown, DOX‐treated mice exhibited a significantly reduced tumor growth (Figure 4Q). Tumor volume and tumor mass were significantly smaller in the DOX‐treated mice expressing the shPDAP1 compared to control groups (‐DOX, shScrambled) at the endpoint (24 days post treatment) (Figure 4Q–S).

*Ex vivo* analysis of xenograft tumors (*n* = 7/group) revealed enhanced lipid‐ROS in PDAP1‐depleted tumors as determined by 4HNE protein adducts, but not of cleaved PARP (Figure 4T), suggesting that KD of PDAP1‐induced ferroptosis not only in vitro but also in vivo. Importantly, the PDPA1‐KD tumors were enriched in the optimized ferroptosis versus apoptosis biomarkers that we discovered (Figure 2F) as determined by qPCR (Figure 4U; Figure S5J, Supporting Information), thus further demonstrating the robustness and reliability of the identified biomarkers. Collectively, these results suggest that PDAP1, which belongs to the “JB2 Up” subset, is a ferroptosis suppressor in basal‐like breast cancer and a potential therapeutic target.

### 2.6 Transcriptomic Analysis of JB2‐ and JB6‐Induced Ferroptotic and Apoptotic Responses

**Figure 5:**
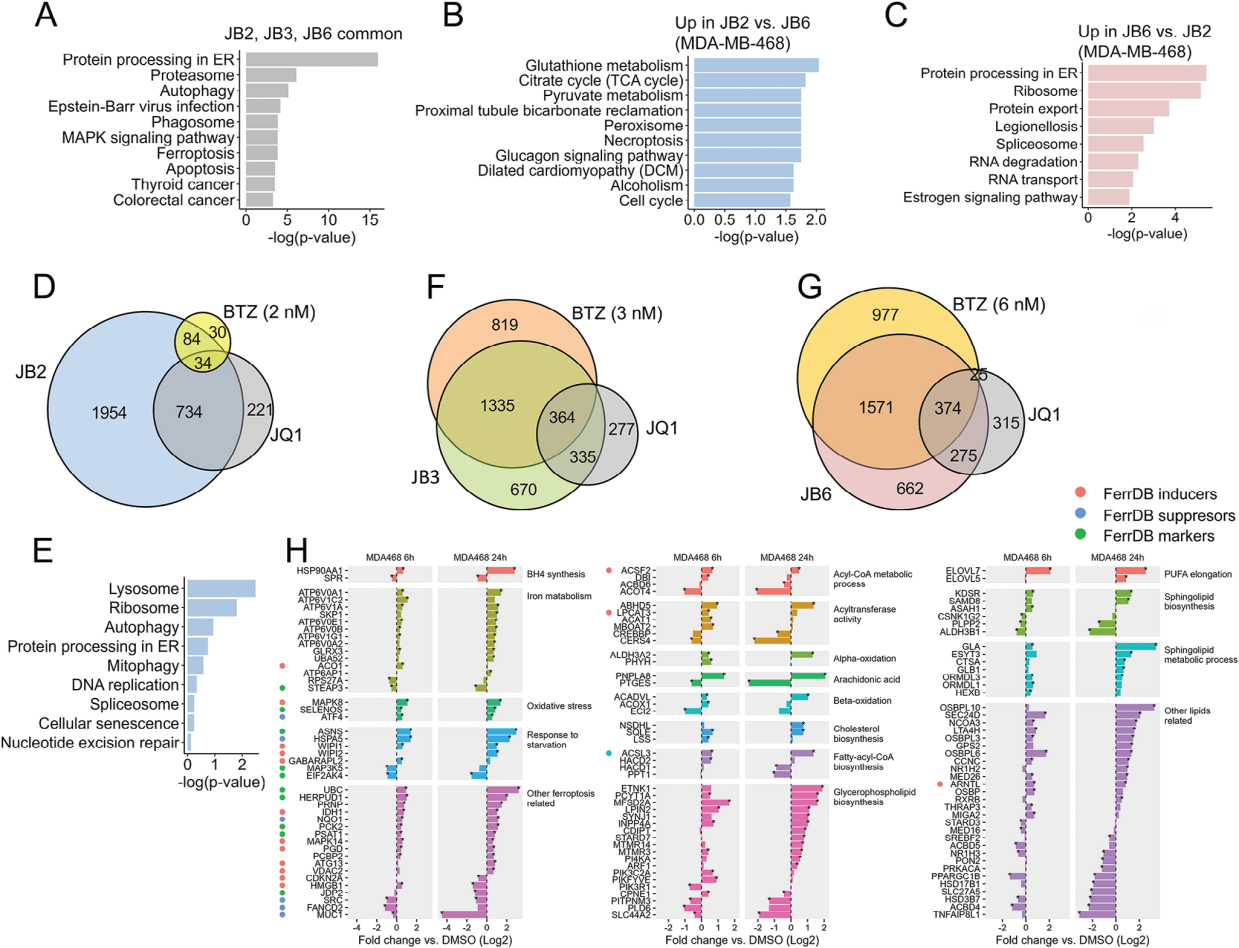
Transcriptomic analysis of the ferroptosis (JB2)‐to‐apoptosis (JB6) transition landscape. (A) KEGG pathways enrichment for upregulated genes by all three JB combinations versus DMSO in both MDA‐MB‐468 and HCC70. (B,C) KEGG pathways enrichment for genes upregulated in JB2 versus JB6 (B) or JB6 versus JB2 (C) in MDA‐MB‐468. (D,F,G) Venn diagram of significantly upregulated genes in MDA‐MB‐468 cells in response to JB2 (D), JB3 (F), or JB6 (G) and their single components versus DMSO. (E) KEGG pathways enrichment for the genes significantly upregulated only in JB2, and not in JQ1 or in B2, versus DMSO in both MDA‐MB‐468 and HCC70. (H) Fold change in gene expression (log2) between JB2 and DMSO in MDA‐MB‐468 in 6 and 24 h. Shown are JB2 exclusive genes relevant to ferroptosis or lipid metabolism. “*” marks statistical significance (FDR < 0.05). Red, blue, and green dots indicate known ferroptosis inducers, suppressors, and markers, respectively, taken from FerrDB.

The established JB2 (ferroptosis)‐to‐JB6 (apoptosis) transcriptomic landscape was used to gain mechanistic insights into each death module. To that end, we compared the DEGs induced by JB2, JB3, and JB6 versus DMSO control. As shown in Figure 1M, ≈80% of the DEGs are common to the three JB‐combinations induced death states, and pathway enrichment analysis (KEGG) of these common genes revealed enrichment of ER stress and the proteasomal pathways in both MDA‐MB‐468 and HCC70 cells (**Figure** **5**A). Enrichment analysis of the upregulated genes in ferroptosis versus apoptosis (JB2 vs JB6) revealed high enrichment of glutathione metabolism and the TCA cycle in MDA‐MB‐468 (Figure 5B) and of oxidative phosphorylation in HCC70 (Figure S6A, Supporting Information). Protein processing in the ER, and ribosome‐related genes were upregulated in JB6 versus JB2 (Figure 5C, Figure S6A, Supporting Information) in both cell lines.

Further analysis of the DEGs of each combination (JB2, JB3, JB6) compared to those of the single drugs revealed robust transcriptomic changes of JB2 in both MDA‐MB‐468 and HCC70 cells (Figure 5D–G; Figure S6B–D, Supporting Information), consistent with their synergy.^[^ ^8a^ ^]^ The JB2 upregulated genes versus single drugs were enriched for lysosomal components, autophagy, and ribosome (Figure 5E). In total, 1872 DEGs were obtained in both cell lines in response to the JB2 combination (vs JQ1 and 2 nM BTZ) at 6 or 24 h post‐treatment (Figure S6D, Supporting Information). Many of these genes were associated with the ferroptotic response of JB2 and included ferroptosis inducers, suppressors, and markers,^[^ ^33^ ^]^ as well as genes known to be involved in ferroptosis‐related processes, such as iron metabolism, TCA cycle, lipid metabolism, and lysosomal function (Figure 5H).

### 2.7 Lysosomal Function is Associated with Ferroptosis

**Figure 6:**
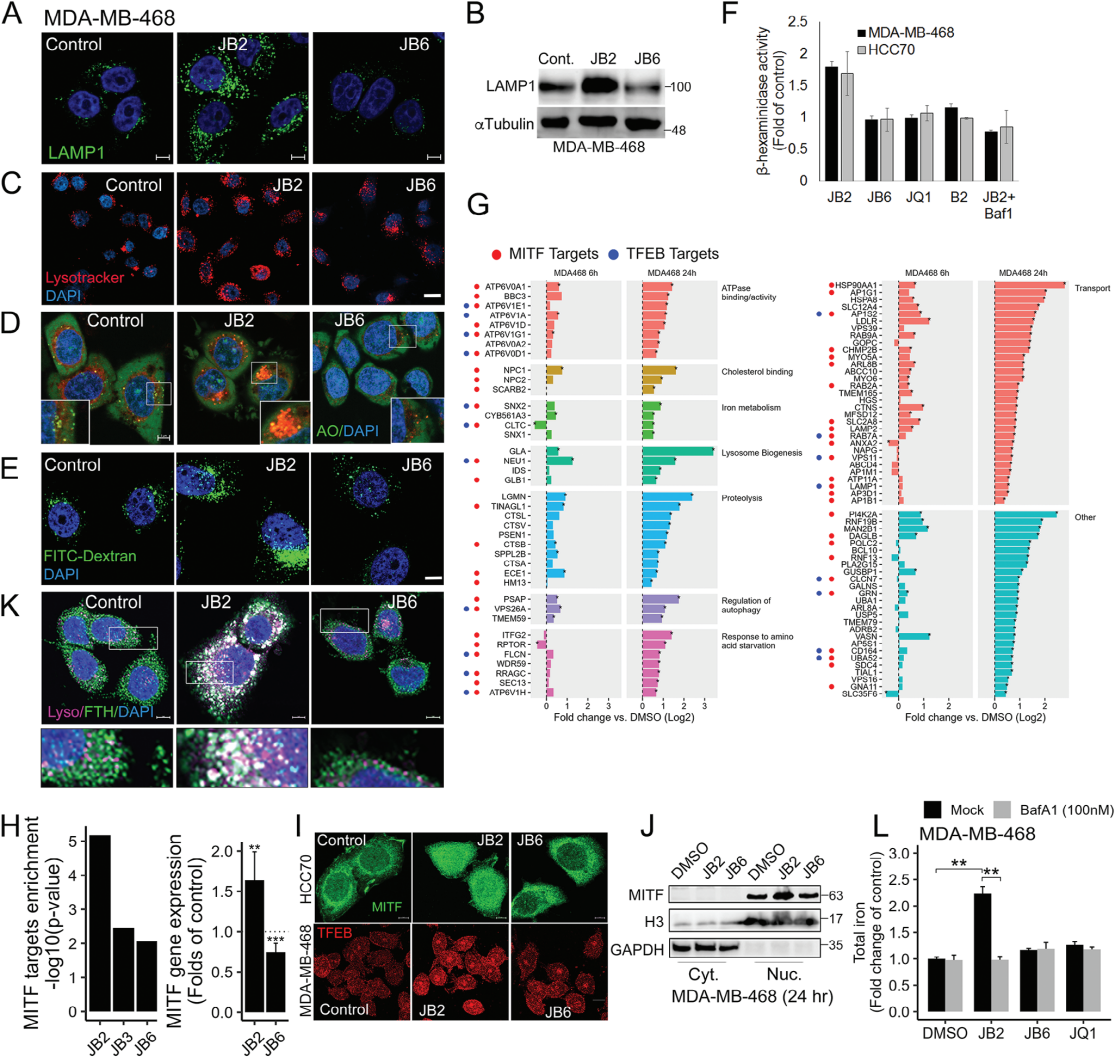
Ferroptotic response is associated with enhanced lysosomal activity and ferritinophagy. (A,B) The level of LAMP1 protein in MDA‐MB‐468 cells treated with the indicated drug combinations for 24 h was assessed by immunofluorescence (IF), Scale bar, 5 µm (A), and by WB (B). (C–E) MDA‐MB‐468 cells were treated with the indicated drug combinations and stained with lysotracker (C) acridine orange (AO) (D) or FITC‐dextran (1 mg ml^−1^)(E). Shown are representative confocal images. Scale bar, 5 µm (D,E), 10 µm (C). (F) MDA‐MB‐468 and HCC70 cells were treated with the indicated drug combinations in the absence or presence of bafilomycin A1 (Baf‐1a, 120 nM) for 24 h. Lysosomal activity was assessed by β‐hexosaminidase activity in cell lysates. Relative activity in treated versus untreated samples was measured and mean values ± SD from two independent experiments are shown. (G) Fold change in gene expression (log2) of lysosome related genes in JB2 (6 and 24 h) versus DMSO in MDA‐MB‐468 cells, measured by RNAseq. `*` indicates significantly regulated genes in JB2 versus DMSO, evaluated by Limma. (H‐J) MITF targets over‐representation in the RNAseq was determined by ChEA3, using the ReMap dataset. Shown is the p‐value of the over‐representation, adjusted for multiple testing (H, left). The effect of JB2 and JB6 on MITF mRNA level was measured by qPCR, 6 h post‐treatment (4 repeats, *p*‐values evaluated by one‐sample t‐test) (H, right). Nuclear localization of MITF or TFEB was assessed 24 h post JB2 or JB6 treatment by either IF analysis, Scale bar, 10 µm, (I), or by cell fractionation (J). Histone 3 (H3) and GAPDH were used as loading control for nuclear (Nuc) or cytosolic (Cyt) fractions (J). (K) Accumulation of ferritin in the lysosome of JB2‐treated MDA‐MB‐468 cells at 24 h as shown by co‐staining of ferratin and lysotracker. Scale bar, 5 µm. (L) Blocking the lysosomal activity by Baf‐1 (100 nM) suppressed the increase in iron levels in JB2‐treated MDA‐MB‐468 cells. Baf‐1 was incubated with the indicated drugs for 24 h and intracellular iron levels were measured by colorimetric assay. Two independent experiments were performed; Shown are mean values ± SD relative to the control of a representative experiment (done in duplicates). *p*‐values are evaluated by t‐test. **p‐value < 0.01, ***p‐value < 0.001.

The significant enrichment for lysosomal genes in JB2‐treated cells (Figure 5E) was associated with an increased level of lysosomal characteristic markers and staining, including the ‐lysosomal‐associated membrane protein 1‐(LAMP1) (**Figure** **6**A,B; Figure S7A, Supporting Information), enhanced LysoTracker staining (Figure 6C; Figure S7B, Supporting Information), increase in acridine orange (AO) acidification as reflected by the red perinuclear spotted signals (Figure 6D),^[^ ^34^ ^]^ increased intensity of perinuclear punctate structures labeled by fluorescent dextran (Figure 6E), and increase of β‐hexosaminidase activity, a lysosomal enzyme that catalyzes the hydrolysis of ganglioside monosialic 2 (Figure 6F).^[^ ^35^ ^]^ Collectively, these results indicate that JB2 enhances lysosomal biogenesis and activity.

Among the 96 lysosomal upregulated genes in JB2, 51 are MITF targets and 19 are TFEB targets (Figure 6G). MITF, TFEB, TFEC, and TFE3 belong to the microphthalmia‐associated family of basic helix loop helix (b‐HLH) leucine zipper transcription factors (MiT/TFE) which regulate the expression of lysosomal and autophagy genes and modulate lysosomal response.^[^ ^36^ ^]^ The upregulation of MITF/TFEB‐target genes in JB2 and their enrichment compared to JB6 (Figure 6H, left) was associated with upregulation of MITF expression (Figure 6H, right), and with MITF/TFEB translocation into the nucleus of JB2‐treated cells (Figure 6I,J; Figure S7C, Supporting Information). The enhanced lysosomal activity was accompanied by a robust accumulation of ferritin in the lysosome (Figure  6K; Figure S7B, Supporting Information). This accumulation implies that ferritin is degraded in the lysosome of JB2‐treated cells through ferritinophagy^[^ ^37^ ^]^ to increase the labile iron pool. Indeed, we observed a strong increase in lysosomal iron, as detected by Ferro‐orange staining (Figure S7D,E, Supporting Information) as well as an increase in total iron levels in JB2‐treated cells which was markedly reduced by Bafilomycin A1 treatment (Figure 6L; Figure S7F, Supporting Information), a drug that prevents lysosomal acidification and ferritinophagy. Bafilomycin A1, as well as the lysosomal inhibitor chloroquine, could partially rescue cell viability of JB2‐ but not of JB6‐treated cells (Figure S7G, Supporting Information), highlighting the impact of the lysosome on JB2 response.

### 2.8 Ferroptosis‐Apoptosis Transition is Associated with XBP1, JNK and ATF4/CHOP Activation

**Figure 7:**
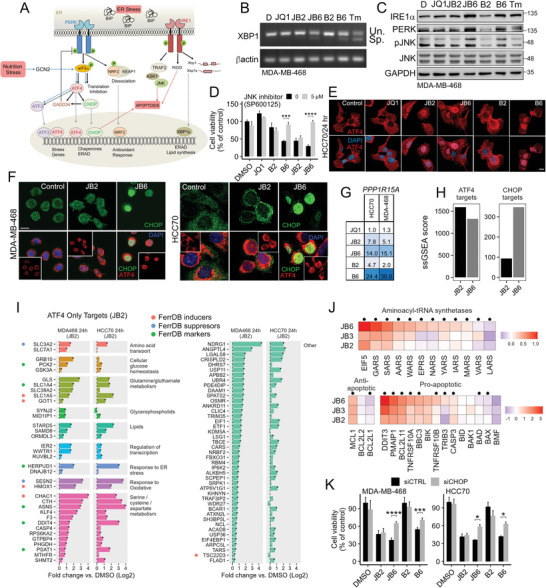
Ferroptosis (JB2)‐to apoptosis (JB6) transition is associated with XBP1, JNK, and ATF4/CHOP activation. (A) Schematic diagram of key pathways in ER stress response. ER stress and/or amino acid deprivation induces the unfolded protein response (UPR) and consequently IRE1α activation. Activated IRE1α cleaves XBP1 mRNA to produce an active XBP1s transcription factor and indirectly activates the ASK1‐JNK axis and apoptotic response. PERK phosphorylates eIF2α to inhibit protein translation, of which ATF4 escapes. ATF4 induces the expression of CHOP and GADD34, a phosphatase that dephosphorylates eIF2α to resume protein translation. ATF4 regulates chaperons, ER‐associated degradation (ERAD), and antioxidant gene transcription. ATF4 also cooperates with CHOP, which plays a role in ER‐stress‐induced apoptosis. (B,C) Activation of IRE1α in response to JB6, B6, or tunicamycin (Tm; 1 µg mL^−1^, a positive control), but not to JB2 treatment, for 24 h in MDA‐MB‐468 cell. (B) XBP1 splicing was detected by RT‐PCR. The unspliced (Un.) and spliced (Sp.) isoforms are shown. (C) WB analysis demonstrates JNK phosphorylation (pJNK) as well as total JNK, IRE1α, and PERK. GAPDH was used for loading control. (D) Inhibition of JNK (by SP600125, 5 µm) partially restored cell viability of B6‐ or JB6‐treated cells. Cell viability was measured by MTT 60 h post‐treatment. Shown are means ± SD of an experiment done in triplicates. P‐values were measured by t‐test. (E,F) Translocation of ATF4 (E) or ATF4/CHOP (F) into the nucleus of HCC70 and MDA‐MB‐468 cells at 24 h post‐treatment with the indicated drugs. Scale bar, 10 µm (for E), 5 µm (for E). (G) PPP1R15A expression levels in response to the applied drugs for 24 h was determined by qPCR analysis and shown as fold of untreated control. The mean values of two independent experiments are shown. (H) Mean fold change of gene expression in JB2/JB6 (24 h) versus DMSO in MDA‐MB‐468 and HCC70 was calculated. Enrichment scores of ATF4 and CHOP targets were measured by ssGSEA. (I) Fold change in gene expression (log2) of ATF4‐only targets in JB2 (24 h) versus DMSO in MDA‐MB‐468 and HCC70 cells, as measured by RNAseq. `*` indicates significantly (FDR < 0.05) regulated genes in JB2 versus DMSO, evaluated by Limma.( J) Heatmap depicts fold change (log2) in gene expression of JB2, JB3, and JB6 versus DMSO, taken from the RNAseq analysis. Top: genes of the aminoacyl‐tRNA synthetases, among the highly enriched pathway in response to CHOP. Bottom: Apoptosis‐related genes. Black dots above heatmaps indicate known CHOP targets. Scale bars indicate log2 fold change versus DMSO. (K) CHOP (DDIT3) was knocked down in MDA‐MB‐468 and HCC70 using siRNA and 24 h later the cells were treated with the indicated drug combinations. Cell viability was measured by MTT 60 h later. Shown are means ± SD of two independent experiments done in duplicates. *p*‐values were measured by t‐test. *p‐value < 0.05, ***p‐value < 0.001, ****p‐value < 1×10^−4^.

The enrichment of ER‐associated pathways was obtained in response to JB2, JB3, or JB6 treatments (Figure 5A), most strongly to JB6 (Figure 5C). ER‐stress response was reported to be associated with apoptosis, ferroptosis, and their crosstalk,^[^ ^4^, ^38^ ^]^ and is known to be induced by proteasomal inhibition. Bortezomib was reported to induce accumulation of misfolded proteins in the ER,^[^ ^39^ ^]^ depletion of intracellular amino acids, and activation of GCN2 (general control nonderepressible 2), which in turn phosphorylates the eukaryotic translation initiation factor 2 (eIF2α) to increase ATF4 translation^[^ ^40^ ^]^ (**Figure** **7**A).

ER stress commonly triggers the unfolded protein responses (UPR) through three main pathways; the IRE1α (inositol‐requiring protein 1α)‐XBP1 (X box binding protein 1) pathway, PERK (PKR‐like ER kinase)‐ATF4, and the ATF6 (activating transcription factor 6) pathway^[^ ^41^ ^]^ (Figure 7A). To evaluate the role of ER‐stress in ferroptotic/apoptotic (JB2/JB6)‐death responses, we examined different arms of the UPR. As shown, JB6 and B6 (6 nM, BTZ), but not JB2, enhanced the RNase activity of IRE1α as indicated by mRNA processing of the unspliced XBP1 into an active spliced isoform (Figure 7B).^[^ ^42^ ^]^ Likewise, JNK phosphorylation (Figure 7C), which is implicated in apoptosis execution downstream of IRE1α,^[^ ^42^ ^]^ was also increased in response to JB6/B6 treatment concomitant with an increase in IRE1α and PERK levels (Figure 7C), while JNK inhibitor (SP600125) substantially restored cell viability of B6/JB6‐ but not of JB2‐treated cells (Figure 7D).

ATF4, a master regulator of stress response, is activated downstream of PERK or GCN2 kinase, which both phosphorylate eIF2α to repress global protein translation and concomitantly enhance translation of a subset of stress‐related mRNAs, including *ATF4* (*CREB2*).^[^ ^43^ ^]^ Cellular fractionation showed enhanced translocation of ATF4 into the nucleus of both JB2‐ and JB6‐treated cells (Figure S8A,B, Supporting Information). The nuclear localization of ATF4 was detected earlier in JB6‐treated cells (at 6 h) (Figure S8C, Supporting Information) and later in JB2‐treated cells (24 h) (Figure 7E), suggesting that both ferroptotic (JB2) and apoptotic (JB6) responses are associated with ATF4 activation.

ATF4 induces transcription of genes involved in amino acid metabolism, glutaminolysis, autophagy, and oxidative stress, and it also regulates the expression of the proapoptotic transcription factor CHOP (GADD153/DDIT3).^[^ ^44^ ^]^ Importantly, CHOP was strongly detected in the nucleus of JB6‐ but not of JB2‐treated cells (Figure 7F; Figure S8D, Supporting Information), consistent with their apoptotic response.

ATF4 and CHOP cooperate to induce the expression of GADD34 (PPP1R15A) and upregulation of GADD34 (growth arrest and DNA damage‐inducible 34). GADD34 dephosphorylates eIF2α to increase protein synthesis which leads to the overloading of unfolded proteins within the ER and apoptotic cell death.^[^ ^45^ ^]^ We observed strong induction of PPP1R15A expression in JB6‐ versus JB2‐treated cells (Figure 7G), concomitant with a decrease in eIF2α phosphorylation (Figure S8E, Supporting Information). Single‐sample GSEA (ssGSEA) analysis of the combined DEGs in MDA‐MB‐468 and HCC70 revealed a slightly higher enrichment of ATF4‐only target genes^[^ ^45^ ^]^ in JB2, but much stronger enrichment of CHOP‐target genes in JB6 (Figure 7H), implying that ATF4/CHOP activation is associated with ER‐stress induced apoptosis, consistent with the nuclear translocation of CHOP (Figure 7F; Figure S8D, Supporting Information). Indeed, the expression levels of many ferroptosis regulators, which are ATF4 targets, were increased by JB2 (Figure 7I), while translation‐related genes, mainly those involved in tRNA synthesis, which are known ATF4/CHOP targets,^[^ ^45^ ^]^ were mainly increased in JB6 (Figure 7J). Furthermore, known pro‐apoptotic CHOP targets (*BCL2L11* [BIM], *BBC3* [PUMA], *PMAIP1* [NOXA], *TRIB3*)^[^ ^46^ ^]^ were also upregulated in JB6‐treated cells (Figure 7J; Figure S8F, Supporting Information), highlighting the impact of CHOP on the apoptotic response. Indeed, CHOP knockdown by siRNA (Figure S8G, Supporting Information) could partially restore cell viability following treatment by JB6/B6, but not JB2 (Figure 7K). Likewise, Pioglitazone, which inhibits ER stress ,^[^ ^47^ ^]^ partially rescued the viability of JB6‐ but not of JB2‐treated cells (Figure S8H, Supporting Information) and concomitantly attenuated translocation of CHOP to the nucleus (Figure S8I, Supporting Information).

Collectively, we found that ATF4 is activated both in ferroptosis and apoptosis, possibly in different kinetics, but the concomitant activation of XBP1, JNK, and CHOP switches to chronic ER stress and apoptotic response.

### 2.9 Ferroptotic Response is Associated with Enhanced Glutaminolysis and TCA Cycle

Several ATF4 target genes, which were upregulated in JB2, regulate glutamine transport or metabolism (Figure 7I), including the two glutamine transporters SLC38A2 and SLC1A5 and the glutamate transporter SLC1A4.^[^ ^48^ ^]^ Importantly, glutaminase 1 (GLS), which converts glutamine to glutamate was also upregulated in JB2‐treated cells (Figure 7I), implying that glutamine metabolism plays an important role in the JB2 response.

**Figure 8:**
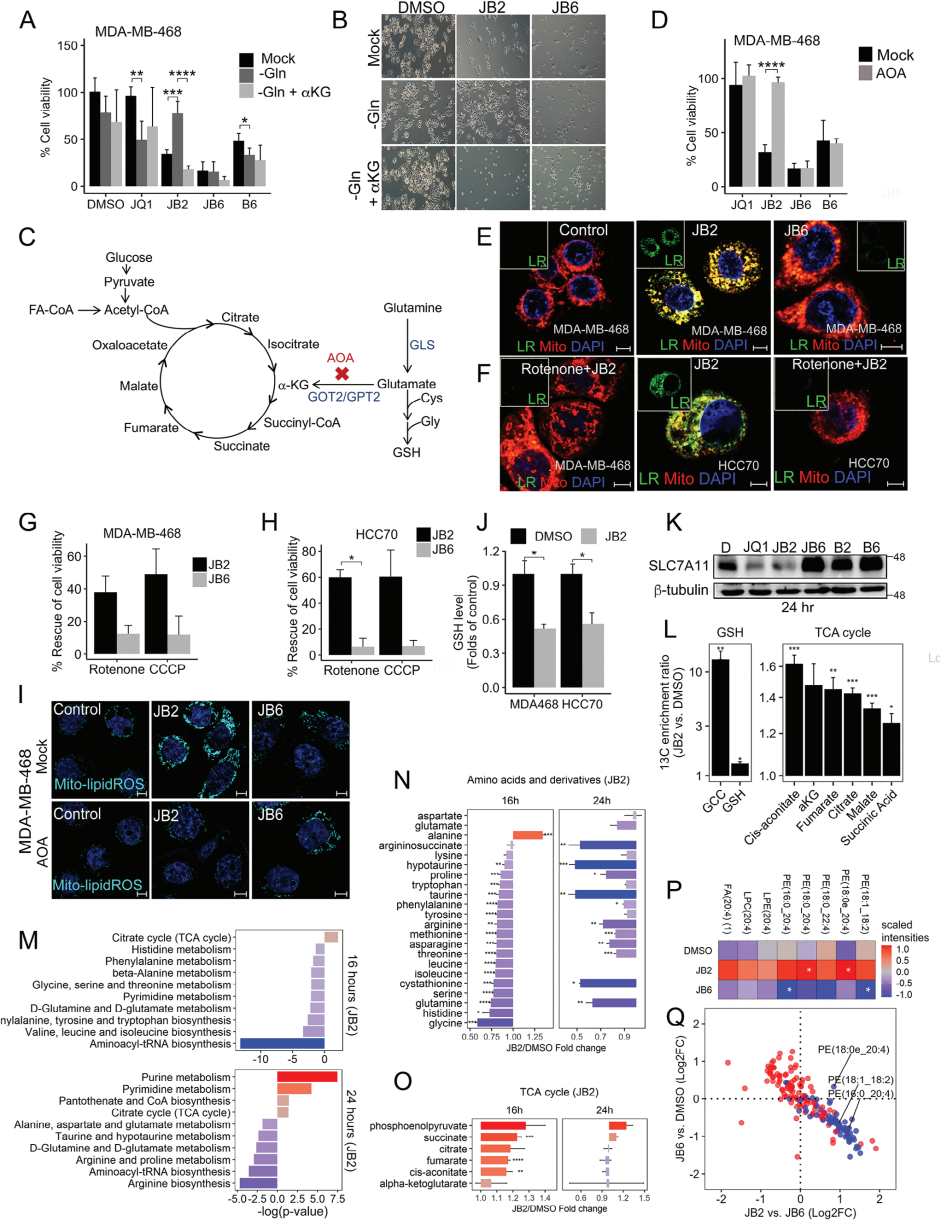
Ferroptotic response of JB2 is associated with metabolic shifts. (A–D) Glutaminolysis is necessary for JB2 mediated ferroptosis. (A) MDA‐MB‐468 cells were treated with the indicated drugs in either normal (Mock), glutamine‐free media (‐Gln), or with glutamine‐free media plus 5 mM α‐ketoglutarate (‐Gln+ αKG). Cell viability was measured after 72 h by MTT and is shown relative to control (%). Mean values ± SD from two independent experiments done in duplicates. P‐values measured by t‐test. (B) Representative images of cells described in A, 72 h post‐treatment. Scale bar, 50 µm. (C) Chart pathway showing the TCA cycle together with the anaplerotic reaction mediated by transaminases such as GOT2 and GPT2, which convert glutamate to αKG using oxaloacetate/pyruvate, and can be blocked by aminooxyacetic acid (AOA). Acytel‐coA is also fueling the TCA cycle through glycolysis and fatty acid oxidation. (D) Cells described in A were treated with the indicated drugs in the absence or presence of AOA inhibitor (0.25 mM). Cell viability was measured 72 h later by MTT and is shown relative to control (%). Mean values ± SD from two independent experiments done in duplicates. P‐values measured by t‐test. (E,F) Blocking the mitochondria ETC rescued JB2‐induced ferroptosis. Representative confocal images of MDA‐MB‐468 or HCC70 cells treated with the indicated drugs with or without rotenone for 16 h. Cells were stained with Mitotracker (red) and C11‐BODIPY (green oxidized). Scale bar, 5 µm. (G,H) Cells described in A were treated with the indicated drugs in the absence or presence of rotenone or CCCP in MDA‐MB‐468 (G) and HCC70 (H). Cell death was measured after 60 h by trypan blue exclusion. Mean values ± SD of % rescue of cell death induced by the JB combinations, from two independent experiments are shown. *p*‐values measured by t‐test. (I) Enhanced mitochondria lipid peroxidation in JB2 cells. MDA‐MB‐468 cells treated with drugs as indicated in the presence or absence of 0.5 mM AOA for 16–18 h, stained with MitoPeDPP probe to detect lipid peroxidation within mitochondria membrane. Shown are representative confocal images. Scale bar, 5 µm. (J,K) JB2 treatment decreased the intracellular level of GSH (J) and the protein level of the xCT subunit SLC7A11 (K) in MDA‐MB‐468 (J,K) or HCC70 (J). Cells were treated with the indicated drugs and 24 h later the level of SLC7A11was assessed by WB (K), while GSH levels were measured by colorimetric assay and are shown relative to control (J). Shown are mean values ± SD from two independent experiments. P‐values measured by t‐test. (L) Gluthamine‐C^13^ tracing in GSH and TCA cycle metabolites. Shown is the total carbon intensity in JB2 versus DMSO of all +2 traces and above. The sum of intensities is weighted (each intensity is multiplied by the number of marked carbons). Shown is the mean ± SD of 4–5 repeats. P‐values were determined by t‐test, comparing JB2 (5 repeats) to DMSO (4 repeats). (M) A polar metabolite screen was performed in JB2 and DMSO. KEGG pathways enrichment for metabolites was performed using Metaboanalyst.Ca. (N,O) Plots show the fold change in normalized intensities of metabolites in JB2 versus DMSO (3–5 individual repeats). *p*‐values were determined by t‐test, comparing JB2 to DMSO. P,Q) Lipidomics profile was performed for JB2, JB6, and DMSO control‐treated MDA‐MB‐468 cells (*n* = 3 independent repeats). (P) Heatmap represents the ferroprosis‐related lipids, 24 h post‐treatment (scaled lipid levels). A T‐test was used to evaluate p‐values. (Q) Fold change in phosphatidylethanolamine (PE, blue dots) and phosphatidylcholine (PC, red dots) in JB6 versus JB2 and DMSO, 24 h post‐treatment. Labeled are lipids relevant to ferroptosis. *p‐value < 0.05,**p‐value < 0.01, ***p‐value < 0.001, ****p‐value < 1×10^−4^.

To assess the impact of glutamine on death response, we incubated MDA‐MB‐468 (**Figure** **8**A) or HCC70 cells (Figure S9A, Supporting Information) in glutamine (Gln)‐free media and assessed cell viability in response to JB2 or JB6 treatment. We found that Gln‐depleted media specifically abrogated JB2‐induced death, while the addition of α‐Ketoglutarate (α‐KG) restored cell death in Gln‐free media (Figure 8A,B; Figure S9A, Supporting Information). These results suggest that α‐KG, a TCA cycle intermediate, which can be produced from glutamate through glutaminolysis,^[^ ^49^ ^]^ and supports oxidative phosphorylation (OXPHOS), plays a role in the JB2‐ferroptotic response. Indeed, inhibition of α‐KG production from glutamate using the glutamate transaminase inhibitor, aminooxyacetate (AOA) (Figure 8C,D; Figure S9B, Supporting Information)^[^ ^50^ ^]^ fully suppressed cell death in response to JB2, but not to JB6.

α‐KG participates in the TCA cycle, which is highly enriched in the JB2 versus JB6 transcriptomic response (Figure 5B) and is also required for electron transport through the mitochondrial electron transfer chain (ETC). We, therefore, evaluated the effects of the ETC inhibitors, Rotenone, a mitochondrial complex I inhibitor, or the mitochondrial uncoupler CCCP (carbonyl cyanide m‐chlorophenyl hydrazine) (Figure 8F–H; Figure S9C, Supporting Information),^[^ ^51^ ^]^ on JB2‐death response. We found that both ETC inhibitors attenuated the death response of JB2 but not of JB6 or B6. Furthermore, we observed extensive colocalization between oxidized BODIPY‐C11 and MitoTracker in JB2‐ but not in JB6‐treated cells (Figure 8E), which was completely abolished in the presence of rotenone (Figure 8F). AOA treatment also reduced the level of mitochondrial lipid ROS in JB2‐treated cells (Figure 8I). Importantly, MitoTracker staining revealed a unique morphology of swollen mitochondria in JB2‐treated cells (Figure S9D, Supporting Information), a characteristic feature of ferroptotic cells.^[^ ^11^ ^]^ Collectively these results suggest that JB2‐cell death is mediated by extensive accumulation of lipid ROS in the mitochondria due to a metabolic switch associated with glutamine‐driven OXPHOS. These results are consistent with previous reports demonstrating the role of glutamine on ferroptosis through the TCA cycle.^[^ ^51^ ^]^

In addition to the anaplerotic role of glutamine in the TCA cycle, glutamine/glutamate are required for biosynthesis of glutathione, for cystine import via the System xc^−^, for synthesis of several nonessential amino acids (alanine, proline, aspartate, asparagine, and arginine), as well as of pyrimidines, purines, hexosamines, and NAD.^[^ ^52^ ^]^ Importantly, we observed significant decreased levels of GSH in JB2‐treated MDA‐MB‐468/HCC70 cells (Figure 8J) concomitant with a substantial decrease in SLC7A11 protein level (a subunit of System xc^−^) (Figure 8K), which both can contribute to ferroptosis execution.

The impact of glutamine on the TCA cycle and GSH biosynthesis was further corroborated by ^13^C‐glutamine tracing experiment. MDA‐MB‐468 were incubated with ^13^C‐glutamine as a tracer and the levels of ^13^C‐labeled TCA intermediates were examined 16 h post JB2 treatment. We observed significant ^13^C‐labeling of the TCA cycle intermediates succinate, malate, and fumarate (M4) as well as citrate and cis‐aconitate (Figure 8L, S9E). We also observed the labeling of glutathione (GSH) and gamma‐glutamylcysteine (GCC; a GSH biosynthesis intermediate) (Figure 8L; Figure S9E, Supporting Information). Collectively, these results demonstrate the critical role of glutamine in JB2‐induced ferroptosis.

### 2.10 Metabolic State of Ferroptosis and its Transition into Apoptosis is Associated with PE/PC Switch

To characterize the effects of JB2 on cellular metabolism, we performed metabolomics and lipidomic profiling of MDA‐MB‐468 cells treated with JB2 or JB6 for 16 and 24 h (Tables S3,S4, Supporting Information). We observed significant upregulation of purine and pyrimidine metabolites and the TCA‐cycle intermediates in response to JB2 (Figure 8M,O), concomitant with a remarkable downregulation of amino‐acids and derivates at both time points, and further robust reduction in glutamine, argininosuccinate, cystathionine, taurine and hypotaurine at 24 h (Figure 8N). Hence, the ferroptotic (JB2)‐death response was associated with global amino‐acids deprivation, and predominantly affected cysteine, glutamine, and arginine metabolism. Although cysteine levels were not measured, cystathionine, an intermediate of cysteine biosynthesis from homocysteine and serine through the transsulfuration pathway^[^ ^53^ ^]^ as well as hypotaurine and taurine, which are generated by the cysteine dioxygenase pathway,^[^ ^54^ ^]^ were markedly reduced. Argininosuccinate, an intermediate in arginine biosynthesis from citrulline and aspartate in the urea cycle,^[^ ^55^ ^]^ was also reduced (Figure 8N).

Similar to JB2‐treated cells, we also observed a global reduction in amino acid levels in JB6‐treated cells (Figure S9F, Supporting Information) possibly due to the strong inhibition of the proteasome (Figure 1J). However, unlike JB6, upregulation of alanine was detected only in JB2‐treated cells, consistent with the enhanced glutaminolysis and glutamate transamination, which can produce alanine from pyruvate by glutamate pyruvate transaminase (GPT).^[^ ^56^ ^]^

As ferroptosis is mediated by lipid peroxidation of PUFAs,^[^ ^57^ ^]^ and JB2 modulates the expression of lipid metabolic genes (Figure 5H), we performed lipidomic profiling of JB2‐ and JB6‐treated cells (Table S4, Supporting Information). As seen in Figure 8P, JB2 treatment increased the level of key lipid species associated with ferroptosis execution, including PE‐containing arachidonic acid (C20:4), linoleic acid (C18:2), and adrenic acid (C22:4),^[^ ^8b^ ^]^ whereas none of these lipids were increased in JB6. The level of PE (18:0/20:4), which is highly susceptible to ferroptosis‐driven oxidation,^[^ ^58^ ^]^ was significantly increased in JB2‐treated cells (Figure 8P). Moreover, an assessment of PE/PC species indicated that the ferroptosis (JB2)‐to‐ ‐apoptosis (JB6) transition was coupled to a global shift from PE to PC (Figure 8Q). Together these observations highlight the robust impact of JB2 on metabolic hallmarks of ferroptosis and demonstrate the distinct metabolic switch associated with JB2 compared to JB6.

## 3 Conclusion

In this study, we used a unique synthetic lethal drug combination targeting BRD4 and the proteasome to induce ferroptosis in basal‐like breast cancer. Subsequently, we established a concentration gradient and systematically reprogrammed a gradual phenotypic, transcriptomic, and metabolomic transition between ferroptosis and apoptosis (Figure 1).

The integrated omics approach together with mechanistic studies highlighted several key principles related to the three death modules, ferroptosis (JB2), apoptosis (JB6), and the intermediate “ferroapoptosis” (JB3). First, the transcriptomic profile of these JB2‐, JB3‐, and JB6‐induced death states is largely overlapping (≈80%), suggesting that they share a common stress response. Nevertheless, each manifests unique transcriptomic changes to induce a selective response (Figure 5A–C; Figure S6A, Supporting Information). Second, the synergistic effect of drug combination is associated with robust transcriptomic landscape reprogramming as obtained for JB2 (Figure 5D; Figure S6B,D, Supporting Information). Third, the different death pathways are associated with differential activities in three cellular organelles, the ER, the lysosome, and the mitochondria (Figure 6, 7, 8). Fourth, the transition between death pathways can be mediated by specific transcription factors and is associated with metabolic switch (Figure 6H and Figure 7H, Figure 8Q).

The GGS, which was generated from the DEGs in the continuous JB2 (ferroptosis)‐to‐JB6 (apoptosis) transcriptomic landscape, is unique, unbiased, and robust. First, it accurately classifies ferroptosis from apoptosis responses of various FINs and AINs across multiple lineages (Figure 2C), despite the specific ferroptotic response of JB2 in TNBC,^[^ ^8a^ ^]^ suggesting that it represents the core gene set downstream of a convergence point of multiple FINs. Second, it was used to optimize a set of selective ferroptosis biomarkers to distinguish ferroptosis from apoptosis (Figure 2D,E). Third, a subset of GGS, the (“JB2 Up”) is associated with poor prognosis and chemotherapy resistance in breast cancer, particularly in TNBC (Figure 3), and thus can be used for patients’ stratification and treatment decisions.

The optimized biomarkers, the 26 “Gradient derived” and 15 “Dataset derived” displayed very similar classification accuracy of ferroptosis and apoptosis, despite their different source and biased/unbiased nature (Figure 2D). These biomarkers were combined into a list of 24 top‐ranked genes which were robustly validated in vitro and in vivo by qPCR, and further assessed in the 45 public datasets and the 5‐validation datasets of FINs in basal‐like breast cancer cells (Figure 2F–H). This rigorous validation convincingly highlights the strength of these biomarkers as reliable transcriptomic tools to distinguish between ferroptosis to apoptosis death modules. These robust transcriptomic biomarkers might be used together with TfR1 immunostaining which was shown as a powerful approach to distinguish ferroptosis from apoptosis.^[^ ^59^ ^]^ Nevertheless, considering the different kinetic responses of various ferroptosis inducers and the multiple mechanisms to induce ferroptosis (Figure S3C, Supporting Information), we expect a variable transcriptomic response for each individual biomarker, but concurrently, a significant enrichment of the 24 biomarkers as an entire set in a given time window (Figure 2I).

We found that the lysosomal pathways are specifically enriched in JB2 (Figure 6), and that MITF and TFEB, which are involved in lysosomal biogenesis were upregulated by JB2. Concomitantly, our RNAseq data indicate that *c‐MYC* was downregulated, and previous reports suggest that MYC competes with MITF/TFEB and suppresses lysosomal biogenesis and autophagy,^[^ ^60^ ^]^ suggesting that the MYC/MITF switch may attribute to lysosomal activation in JB2.

The ER processing/stress pathway was found to be commonly enriched by JB2‐induced ferroptosis and JB6‐induced apoptosis, albeit more strongly in apoptosis (Figure 5A,C, Figure 7). Activation of ATF4 (Figure 7E; Figure S8A–C, Supporting Information), a major transcription factor of the integrated stress response,^[^ ^16^ ^]^ was also observed by the two death modules. Nevertheless, the JB2 (ferroptosis)‐to‐JB6 (apoptosis) transition was coupled to XBP1, JNK, and ATF4/CHOP activation (Figure 7B–F). Activation of CHOP by ER stress and its further involvement in ER oxidation and apoptotic response was demonstrated by previous studies.^[^ ^61^ ^]^ ATF4‐CHOP overexpression was proposed to increase protein translation by upregulating ribosomal protein expression, which consequently caused to increase in protein synthesis, ATP depletion, oxidative stress, and apoptotic cell death.^[^ ^45^ ^]^ CHOP was also proposed to increase oxidative damage within the ER through upregulation of the ER oxidase 1 (ERO1), which activates the inositol 1,4,5‐triphosphate receptor (IP3R) to release calcium and consequently induce apoptotic cell death.^[^ ^61a^ ^]^

The strong activation of ATF4 by both JB6‐ and JB2‐induced apoptosis and ferroptosis was associated with global amino acids deprivation in response to both death pathways (Figure 8N; Figure S9F, Supporting Information). However, several ATF4 target genes that were strongly changed in ferroptosis play roles in glutamine/glutamate transport. Indeed, we found enhanced glutaminolysis and upregulation in TCA cycle metabolites and genes (Figure 5B, Figure 8A,B,L,O, Figure S9A,B,E, Supporting Information). We also found that inhibition of the anapleurotic reactions feeding the TCA cycle or the ETC was sufficient to rescue the cells from ferroptosis (Figure 8A–H), similar to their effects on cysteine deprivation‐induced ferroptosis.^[^ ^51^ ^]^

Our finding that ER stress is associated with both JB2/JB6‐induced ferroptosis and apoptosis, but more prominent in apoptosis, is possibly related to the gradual inactivation of the proteasome activity over the concentration gradient. While high BTZ (6 nM) concentration strongly inhibited the proteasomal activity (Figure 1J) and consequently induced strong ER‐stress response and UPR (Figure 7B,C,E), low BTZ concentration (B2) had significantly weaker effects on the proteasome activity (Figure 1J), ER stress and the overall transcriptome. Nevertheless, in the presence of a low JQ1 dose, both combinations of JB2 and JB6 induced strong stress response reflected by ATF4 activation (Figure 7E; Figure S8A,B,C, Supporting Information).

Importantly, depletion of PDAP1 in B cells also caused a sustained expression of ATF4 and induction of ATF4 stress response transcriptional program,^[^ ^62^ ^]^ and our data showed that PDAP1 depletion in basal‐like breast cancer induced ferroptotic cell death and significantly reduced tumor growth in xenograft model (Figure 4). Knockdown of PDAP1 in colorectal cancer also inhibited tumor growth in PDX model but had a minor effect, if at all, on apoptosis in cultured cells.^[^ ^28a^ ^]^ Likewise, PDAP1 depletion in breast tumors was associated with increased 4HNE staining, but not with cleaved PARP (Figure 4T), despite that the viability of PDAP1‐depleted MDA‐MB‐468 cells in vitro was partially restored by ZVAD (Figure S5C, Supporting Information), implying a possible crosstalk between the two death pathways upon PDAP1 knockdown.^[^ ^63^ ^]^ Although the death module imposed by PDAP1 depletion could be cell type‐specific, we found that PDAP1 knockdown in basal‐like breast cancer cells induced canonical hallmarks of ferroptosis.^[^ ^18^ ^]^ Our mechanistic studies suggest that PDAP1 protects from ferroptosis by positively regulating the AKT‐mTOR‐SRBP1‐SCD1 signaling axis (Figure 4N–P; Figure S5I, Supporting Information). This protective role is associated with the related transcriptomic response of PDAP1 knockdown and GPX4 depletion, as determined by CMAP analysis (Figure S5A, Supporting Information), and thus introduced PDAP1 as a novel ferroptosis regulator. Indeed, PDAP1 is one of the GGS genes and belongs to the “JB2 Up” subset consisting of potential ferroptosis repressors.

In summary, by programming a unique continuous ferroptosis (JB2)‐to‐apoptosis (JB6) landscape, we identified critical molecular switches between these death pathways, and defined a gradient gene set, the GGS, as an excellent predictor for ferroptosis and apoptosis classification. The GGS was also used to optimize a list of reliable, validated ferroptosis biomarkers and to predict chemoresistant tumors. Furthermore, the GGS is a unique source of novel ferroptosis regulators, such as PDAP1, which might be used as targets for cancer therapy.

## 4 Experimental Section

### Drugs and Chemicals

The following drugs and chemicals were used in this study: JQ1 (11187), Bortezomib (BTZ, 10008822), Liproxstatin‐1 (17730), rotenone (13995) and carbonyl cyanide m‐chlorophenyl hydrazine (CCCP, 25458) were purchased from Cayman Chemicals. SP600125 (1496) was from Tocris Bioscience. Imidazole ketone erastin (IKE) was from Taizhou Crene Biotechnology, China. The following were purchased from Sigma: Ferrostatin‐1 (SML0583), zVAD‐FMK (V116), Necrostatin‐1 (N9037), reduced l‐GSH (G6013), 2,2′‐bipyridyl (D216305), staurosporine (S5921), Doxicycline (D9891, D3072) bafilomycin‐A1 (B1793), α‐ketoglutarate (K1128), erastin (E7781), RSL3 (SML2234), FIN56 (SML1740), aminooxyacetic acid (AOA, C13408), pioglitazone (CDS021593), tunicamycin (T7765), paclitaxel (T7402) and chloroquine (C6628).

### Antibodies

The antibodies list is provided in Supplementary Table S5 (Supporting Information).

### Cell Culture

The basal‐like breast cancer cell lines MDA‐MB‐468, HCC70, HCC38, the luminal breast cancer line MCF7, and the human embryonic kidney (HEK) 293T cells were originally obtained from the American Type Culture Collection (USA). MDA‐MB‐468, HCC70, HCC38, and MCF7 were grown in RPMI, while HEK293T cells were grown in Dulbecco's modified Eagle medium (DMEM) (Gibco BRL, USA). Unless otherwise indicated, all cell lines were cultured in a medium containing 10% fetal bovine serum (Gibco BRL, USA), L‐Glutamine (2 mM), and penicillin/streptomycin. Cells were cultured at 37 °C in a humidified incubator of 5% CO_2_. Cell lines were routinely (once a month) checked for mycoplasma using an RT‐PCR.

### Drugs Treatments

JQ1 and Bortezomib (BTZ) were dissolved in DMSO (20 mM stock for JQ1, and 10 mM for BTZ), aliquoted, and stored at −20 °C protected from light. Drugs were routinely applied at a cell density of ≈50% confluency, ≈24 h after cell seeding. BTZ was diluted to 20 µM in DMSO and JQ1 was diluted to 200 µM in RPMI media. Drug combinations were freshly prepared at a final concentration (0.1 µM for JQ1 and 2–6 nM for BTZ) in a growth medium before use, by vigorous vortex (3 min). DMSO concentration was maintained below 0.1%. Importantly, batch effects of JQ1 and BTZ might influence the final concentration for each JB combinations and should be carefully calibrated due to the delicate titration and death response. Ferrostatin‐1 (100 mM stock in DMSO), liproxstatin‐1 (25 mM in DMSO), and zVAD‐FMK (20 mM stock in DMSO) were applied 2 h before and during the drugs treatment at a final concentration of 5–10 µM.

### Drug Response Assays

Various assays were applied in response to drug treatments (cell viability, cell death, lipid peroxidation, qPCR, RNAseq, iron measurements, etc), and were performed at different time points as indicated for each assay and in the corresponding legends. The different time points related to the different kinetics of the various responses, which were calibrated in preliminary experiments. Usually, lipid peroxidation (BODIPY, 4HNE), as well as its effect on other cellular proteins, was measured 16–24 h post‐treatment, while cell death and viability are measured at longer time points, usually 72 h post‐drug treatment.

### Cell Viability and Death Assays

For MTT assay, 5000 cells were seeded in a 96‐well plate and 24 h later treated with the indicated drugs. Cell viability was measured 72 h later using the MTT (M2128, SIGMA) assay as described previously.^[^ ^64^ ^]^ Results were presented as % cell viability of control untreated (DMSO) cells. For the trypan blue exclusion assay, the cell culture media was collected following drug treatment, combined with the trypsinized cells that remained on the plate, and spun down at 1 200 rpm for 5 min. Pellets were resuspended in PBS, mixed 1:1 with trypan blue, and counted by an automated cell counter (Countess‐II, Invitrogen). A fraction of trypan blue negative cells was used to calculate cell viability compared to the control (mock‐treated cells). Cell death was measured by the CellTox‐green Cytotoxicity assay (G8741, Promega) according to the manufacturer instructions. In brief, 5000 cells/well in 96 wells black plate (Greiner, 655 090) were treated with the indicated drugs in the absence or presence of either liproxstatin‐1 or ZVAD for 72 h. CellTox green reagent was diluted 1:1000 in assay buffer and incubated with the cells (100 µl well^−1^). Plates were shaken for 1 min using an orbital shaker and then incubated in the dark at room temperature for 10–15 min. The fluorescence signal was measured by the Infinite 200 PRO Tecan fluorometric microplate reader at 490/525 nm (excitation/emission).

### RNA Extraction and Real‐Time PCR

Total RNA from cell lines was extracted and purified using TRI Reagent (Sigma‐Aldrich). RNA was reverse‐transcribed into complementary DNA (cDNA) using the High‐Capacity cDNA Reverse Transcription Kit (Applied Biosystems; Cat. No. 4368814) with random primers according to the manufacturer's instructions. Real‐time PCR analysis was performed in QuantStudio‐3 Real‐Time PCR system (Applied Biosystems, Thermo Fisher Scientific) using SYBR Green Master Mix reagents (Roche) according to the manufacturer's guidelines. House‐keeping gene β‐acting was used for normalization. The relative levels of mRNA were calculated using the ΔΔ*C*_T_ method. The primers list is provided in Table S6, Supporting Information.

### RNA Sequencing

RNAseq was performed for control (DMSO) basal‐like breast cancer cell lines, for cells treated with JB2, JB3, JB6, or single treatments (B2, B3, B6, JQ1), as well as with ferroptosis inducers (erastin, RSL3). DMSO concentration was similar in all samples. Two‐time points were selected for the RNAseq of JQ1/BTZ/JB2/JB3/JB6 treatments, 6 and 24 h, based on qPCR analysis of well‐known ferroptotic upregulated genes (ASNS, CHAC1, DDIT4)^[^ ^21^ ^]^ concomitantly with cell viability assay. At 24 h no effect on cell viability was observed, but the indicated genes were significantly upregulated, while the earlier time point (6 h) represents the initial transcriptomic response to the treatments. All RNAseq samples were done in independent duplicates. Total RNA was extracted as described above. RNA quality was assessed using the Agilent 4200 TapeStation System (Agilent Technologies, Santa Clara, CA). RNA‐seq libraries were generated by applying a bulk adaptation of the MARS‐seq protocol, as previously described.^[^ ^65^ ^]^ Libraries were sequenced by the Illumina Novaseq 6000 using SP mode 100 cycles kit (Illumina). The mapping of sequences to the genome and generation of the count matrix was performed by the UTAP pipeline (Weizmann Institute). Libraries normalization, filtration of low count genes, and discovery of differentially expressed genes were performed using the edgeR and Limma packages in R. PCA plots and a Venn diagram was generated using the factoextra and eulerr packages in R, respectively. KEGG enrichment analysis was performed using the enrichR package; p‐values for KEGG enrichment analysis were determined by Fisher exact test and adjusted for multiple hypotheses testing by the Benjamini‐Hochberg method. Gene set enrichment analysis (single sample) was performed using the GSVA package. Over‐representation of transcription factor targets was performed using ChEA3.^[^ ^66^ ^]^

### Gene Signatures and Biomarkers

Computational analysis of the RNAseq to produce the gradient gene set, the specific ferroptosis to apoptosis biomarkers, and their validations using UMAP and ssGSEA were all done in R. The computational methods are detailed in the supplementary text.

### Cell lysates and Western Blotting

Cell were lysed in lysis buffer containing 0.5% Triton X‐100, 50 mM Hepes (pH 7.5), 100 mM NaCl, 1 mM MgCl_2_, 50 mM NaF, 0.5 mM NaVO_3_, 20 mM β‐glycerophosphate, 1 mM phenylmethylsulfonyl fluoride, 10 µg ml^−1^ leupeptin, and 10 µg ml^−1^ aprotinin. Freshly isolated tumors were manually chopped with a sterile blade, and 50 mg tissue was homogenized in 400 µl lysis buffer using 4 mm stainless steel beads in a homogenizer device (TissueLyser‐LT, QIAGEN) for 4 min (or until a homogenous solution was obtained). Cell lysates were centrifuged at 14,000 rpm for 15 min at 4 °C, protein concentration of the supernatants was measured by Bradford assay (Bio‐Rad, Hercules, CA). Equal amounts of total protein (30–50 µg per sample) were analyzed by SDS–PAGE (polyacrylamide gel electrophoresis) and Western Blotting (WB) was performed as previously described.^[^ ^67^ ^]^

### Cell Fractionation

Nuclear and cytoplasmic extracts were prepared as follows: 1 × 10^6^ cells were seeded in 10 cm plates for ≈24 h, then treated with drugs for the indicated time periods, washed twice with cold PBS, and lysed with 250 µl of cytoplasmic extract (CE) buffer (0.075% (v/v) NP40, 10 mM HEPES, 60 mM KCl, 1 mM EDTA, 1 mM DTT, 1 mM PMSF, 0.5 mM NaVO_3_, 10 µg ml^−1^ leupeptin, and 10 µg mL^−1^ aprotinin, adjusted to pH 7.6). Samples were centrifuged at 1,000 rpm for 5 min at 4 °C and cytoplasmic fractions were collected from the supernatant. Pellets were washed gently with 250 µl of CE buffer without NP40 and spun again. Nuclear fractions were extracted by nuclear extract (NE) buffer (20 mM Tris HCl, 420 mM NaCl, 1.5 mM MgCl_2_, 0.2 mM EDTA, 1 mM PMSF, 25% (v/v) glycerol, 0.5 mM NaVO_3_, 10 µg mL^−1^ leupeptin, 10 µg mL^−1^ aprotinin, adjusted to pH 8.0). Pellets were resuspended in 100 µL NE buffer containing 400 mM NaCl, vortexed, and incubated on ice for 10 min. The cytoplasmic and nuclear fractions were centrifuged at 14,000 rpm for 15 min at 4 °C. Supernatant from the CE and NE was collected. Protein content was measured by Bradford and samples were analyzed by SDS‐PAGE and WB.

### Fluorescence Staining

Cells grown on coverslips in a 24‐wells plate were treated with drugs as indicated, washed twice with PBS, fixed in 4% paraformaldehyde (PFA), and processed for immunofluorescence (IF) staining essentially as described previously^[^ ^68^ ^]^ using the following IF blocking buffer (10 mM Tris‐Hcl pH 7.5, 150 mM NaCl, 2% bovine serum albumin (BSA), 10% goat serum, 1% Glycine, 0.05%–0.1% Triton‐X‐100).

Live cell imaging was performed with MitoTracker (Red‐CMXRos, Invitrogen) or LysoTracker (Red DND‐99 or Green DND‐26, Invitrogen). Cells grown in a 96‐well glass bottom plate (8000 cells/well) were treated with drugs as indicated, washed with PBS, and incubated in either serum‐free RPMI media containing 70 nM of MitoTracker or in full RPMI (10% FBS) media with 70 nM of LysoTracker. Hoechst (1 mM) was also added to the media and the cells were incubated for 30 or 45 min, respectively, in a tissue culture incubator in the dark. The cells were then washed gently in PBS and incubated with 100 µL of live cell imaging solution (Invitrogen) for analysis by confocal microscopy (LSM800, Zeiss). Co‐staining with LysoTracker of fixed cells was performed by staining with LysoTracker (80 nM) as described above, followed by fixation in 4% PFA and blocking in IF blocking buffer containing 0.05% Triton‐X‐100 as described above.

### Acridine Orange

Cells were incubated in serum‐free RPMI media with 1 µg mL^−1^ Acridine Orange (AO) (ImmunoChemistry Technologies) and 1 mM Hoechst 33 342 (Sigma‐Aldrich) for 30 min. Cells were then washed with PBS incubated with live cell imaging solution and processed by confocal microscope.

### FITC‐Dextran

The polysaccharides dextran conjugated to FITC (FD10S, 10 KDa, SIGMA), which accumulates in the lysosome,^[^ ^69^ ^]^ was incubated (1 mg mL^−1^) with the cells grown on coverslips in complete RPMI media for 2 h. The cells were washed in PBS and incubated in complete RPMI media for an additional 2–3 h, and then treated with drugs as indicated for 24 h. Cells were washed with PBS, fixed with 2% formaldehyde for 25 min at RT, and stained with 1 mM Hoechst in PBS for 5 min. Images were processed by confocal microscope (LSM800, Zeiss).

### Animal Studies

All animal studies were performed according to protocols approved by the Weizmann Institutional Animal Care and Use Committee (IACUC approval numbers 00350122‐2 for the PDAP1 in vivo experiment, and 08311121–2 for the in vivo validation of the biomarkers).

For PDAP1 mouse xenograft models, GFP‐luciferase expressing MDA‐MB‐468 cells were infected with Tet‐pLKO‐shPDAP1 lentivirus, selected with puromycin (1 µg ml^−1^) and then implanted bilaterally into the mammary fat pads (2 × 10^6^ per gland) of the fourth inguinal gland of 5–6 weeks old female athymic nude‐*Foxn1nu* mice. Tet‐pLKO harboring scrambled sequence was used as control. When tumor size reached an average of ≈50 mm^3^, mice were randomized into two groups (*n* = 10 mice per group for shPDAP1, *n* = 5 for scrambled shRNA); control (regular water) and DOX experimental group (2 mg mL^−1^ of doxycycline (D9891, SIGMA) in drinking water, DOX was replaced every 4–5 days). Mouse body weights and tumor size were measured every 3–4 days. Tumor volume was measured by a digital Vernier caliper and calculated according to the (width^2^ × length)/2 formula. Tumor imaging was monitored using Xenogen IVIS Spectrum in vivo bioluminescence. Mice were anesthetized by isoflurane inhalation, and then D‐luciferin (100 µL; 5 mg mL^−1^; Regis Technologies) was intraperitoneally injected. Bioluminescence images were acquired within 10 min after injection by the charge‐coupled device camera of the IVIS instrument with the Living Image 3.0 software (Xenogen Caliper Life Sciences). At the end of the study, mice were sacrificed, and tumors were excised, weighed, and documented using a digital camera.

For *invivo* validation of the selective ferroptosis biomarkers, MDA‐MB‐468 cells were orthotopically implanted into mammary fat pads as described above. When tumor size reached an average of ≈50 mm^3^, mice were randomized into three groups (*n* = 3 mice per group): (1) IKE‐ imidazole ketone erastin (Taizhou Crene Biotechnology, China), 3 days 40 mg kg^−1^, and 80 mg kg^−1^ on the 4th day (2) PTX‐paclitaxel (#1567‐125, Biovision) 20 mg kg^−1^, and (3) Ctrl ‐control. IKE was dissolved in HBSS buffer containing 5% DMSO at pH‐4, and paclitaxel was dissolved in HBSS. Drugs were administered intraperitoneal in a dose of 100 µL per mouse (ctrl group received only HBSS/5% DMSO, pH‐4) and 96 h later mice were anesthetized by intraperitoneal injection with a lethal dose of pental (300 µL, 4 ml kg^−1^). Tumors were excised and immediately processed for RNA purification or WB analysis.

### GSH, Lipid Ros, and Iron measurements

Methods have been previously described,^[^ ^8a^ ^]^ and are included in the Supplementary Methods

### Supplementary Methods

Gene knockdown, Mitochondrial, ER, and lysosomal manipulations, GSH measurements, Lipid ROS measurements, FACS analysis, iron measurements, proteasomal activity assay, iron staining, β‐hexaminidase activity assay, metabolomics, and glutamine tracing are detailed in the supplementary methods section.

### Statistical Analysis

The statistical test used to determine the significance levels for each experiment was done in R and is described in the figure legends. In most figures (unless otherwise mentioned in the figure legends), the means ± sd of the repeats are shown, and a two‐sided t‐test was used to measure statistical significance. The sample size for each experiment is described in the figure legends. *p*‐values lower than 0.05 were considered statistically significant.

## Conflict of Interest

The authors declare no conflict of interest.

## Author Contributions

Y.V. and A.M. contributed equally to this work. Y.V. designed and performed the computational analysis (Figure 1K–M, Figure 2, Figure 3U, Figure 4, Figure 5G,H, Figure 6I,J, Figure S1K,L,S2,S4,S5A,S6, Supporting Information), assisted in writing the manuscript and checked the raw data. A. Maimon designed and performed all the experiments except those mentioned below. V.D. performed the experiments shown in Figure 1A,B, Figure S1A,B, Figure 6A,D,F,K,I, Figure S7A,B, (Supporting Information) and prepared the RNA for RNAseq. H.R assisted with the immunofluorescence experiments shown in Figure 1E, Figure 6I.. I.A. performed the glutamine tracing experiment (Figure 8L). S.M. and M.I. performed the polar metabolites and lipidomics screen. A. Ma'ayan contributed to the discussion and reviewed the paper. F.W. contributed to the discussion and reviewed the paper., E.G., supervised the glutamine tracing experiment. E.R. provided valuable suggestions for the computational studies, and reviewed and edited the manuscript. S.L. designed and supervised the study, contributed to the analysis and interpretation of the data, ensured financial support, was involved in figure preparation, and wrote the manuscript.

## Supporting information

Supporting Information

Supplemental Table 1‐6

## Data Availability

RNA‐seq data is publicly available in the Gene Expression Omnibus (GEO) repository (https://www.ncbi.nlm.nih.gov/geo/), accession number GSE235201. The code used to generate the computational analysis is available through GitHub (https://github.com/SimaLevLab/A‐continuous‐ferroptosis‐to‐apoptosis‐landscape). All other data generated in this study is available upon request from the corresponding author.
