# Supplementary material for: Programming a Ferroptosis‐to‐Apoptosis Transition Landscape Revealed Ferroptosis Biomarkers and Repressors for Cancer Therapy
Source: Adv Sci (Weinh). 2024 Mar 5;11(17):2307263. doi: 10.1002/advs.202307263 (PMC11077643; doi:10.1002/advs.202307263)
Supplement: Supplementary file 1 — Supporting Information [file ADVS-11-2307263-s001.pdf]

ADVANCED SCIENCE
Open Access

Supporting Information

for *Adv. Sci.*, DOI 10.1002/advs.202307263

Programming a Ferroptosis-to-Apoptosis Transition Landscape Revealed Ferroptosis Biomarkers and Repressors for Cancer Therapy

*Yaron Vinik, Avi Maimon, Vinay Dubey, Harsha Raj, Ifat Abramovitch, Sergey Malitsky, Maxim Itkin, Avi Ma'ayan, Frank Westermann, Eyal Gottlieb, Eytan Ruppin and Sima Lev**

Supporting Information

Programming a ferroptosis-to-apoptosis transition landscape revealed ferroptosis biomarkers and repressors for cancer therapy

Yaron Vinik, Avi Maimon*, Vinay Dubey, Harsha Raj, Ifat Abramovitch, Sergey Malitsky, Maxim Itkin, Avi Ma'ayan, Frank Westermann, Eyal Gottlieb, Eytan Ruppin and Sima Lev†*

*These authors contributed equally to this work

†Corresponding author: Sima Lev, Weizmann Institute of Science, Email: Sima.Lev@weizmann.ac.il

Supplementary Methods

Gene knockdown

shRNA lentivirus*–mediated knockdown:* lentiviral vectors encoding shRNAs of PDAP1 and GPX4 were purchased from Sigma (TRCN0000299988 and TRCN0000299984 for PDAP1, TRCN0000046252 for GPX4), and prepared as described in [1].

Tet-ON inducible knockdown*:* The Tet-pLKO-puro lentiviral vector was purchased from Addgene as a gift from Dmitiri Wiederschain (plasmid_#21915; http://n2t.net/addgene:21915; RRID:Addgene_21915). The shRNA oligonucleotides were cloned into the Tet-pLKO-puro vector according to the manufacture instruction.

Oligonucleotide sequence for PDAP1 knockdown (similar to target sequence of TRCN0000299988):

Forward:

5’CCGGCTTTCGAGGAGAGAACGAGAACTCGAGTTCTCGTTCTCTCCTCGAAAGTTTTTG 3’,

Reverse:

5’ AATTCAAAAACTTTCGAGGAGAGAACGAGAACTCGAGTTCTCGTTCTCTCCTCGAAAG 3’.

Oligonucleotide sequence for Scramble (control knockdown):

Forward:

5’ CCGGCAACAAGATGAAGAGCACCAACTCGAGTTGGTGCTCTTCATCTTGTTGTTTTTG 3’

Reverse:

5’ AATTCAAAAACAACAAGATGAAGAGCACCAACTCGAGTTGGTGCTCTTCATCTTGTTG 3’

siRNA knockdown*:* CHOP siRNA (SC-35437, Santa Cruz) was used to knockown CHOP, while a non-targeting siRNA (D-001210-05-05, Dharmacon) was used as control. Transfection of siRNA was performed using DharmaFECT Transfection Reagents (Dharmacon), following the manufacturer instructions.

Mitochondrial, ER and lysosomal manipulations

The mitochondrial electron transport chain blocker rotenone (4 μM), an inhibitor of the mitochondrial complex-I, or the mitochondrial uncoupler carbonyl cyanide m-chlorophenyl hydrazone (CCCP) (4 μM)

were incubated together with JQ1 and BTZ for 60 hr, and their effects on cell death was measured by trypan blue exclusion assay. Where indicate, cells were incubated in glutamine free RPMI media (21870076, Gibco) to induce glutamine deprivation, and α-ketoglutarate (K1128, SIGMA) was added (10 mM) as indicated.

To inhibit glutaminolysis, we used the pan-transaminase inhibitor aminooxyacetic acid (AOA, C13408, SIGMA). Briefly, 5,000 cells were seeded in 96-wells plate and 24 hr later were treated with the indicated drugs in the absence or presence of 0.25 mM AOA for 72 hr. Cell viability was measured by MTT assay.

To inhibit lysosomal activity, we used the vacuolar type H^+-ATPase inhibitor bafilomycin A1 (Baf1), (B1793, SIGMA). Briefly, $1*10^6$ cells were seeded in 10 cm plates and 24 hr later were treated with the indicated drugs in the absence or presence of 120 nm of Baf1. After 24 hr incubation cells were analyzed either for β-hexaminidase activity or for total intracellular iron level using colorimetric assay. For cell viability rescue experiment with bafilomycin, we used low concentration (1nM) due to high toxicity. To inhibit ER-stress, 10 μM pioglitazone (CDS021593, SIGMA) was incubated with JB combination for 72 hr and cell viability was measured by MTT.

GSH measurements

GSH levels were measured by a Glutathione Colorimetric Assay kit (K261, BioVision) according to the manufacturer instructions. Cells were lysed in a GSH assay buffer, and GSH levels were detected by absorbance at 405 nm using a microplate reader (Infinite 200 PRO Tecan). The GSH concentration was calculated according to a standard curve. The values were then normalized to total protein levels in lysate samples. Results were expressed as fold of untreated control.

Lipid ROS measurement

Lipid peroxidation in live cells was detected by the C11-BODIPY 581/591 fluorescence reporter (#27086, CAYMAN). Cells were plated in High-Content Imaging Glass Bottom 96-well Microplates (Cellvis) for ~24 hr and then treated with the indicated drugs. The lipid peroxidation sensor C11-BODIPY (581/591) (7 μM) was added for 30-40 min together with 1 mM Hoechst 33342 (Sigma-Aldrich) in regular RPMI full media. Cumene hydroperoxide (CH, 75 μM preincubation for 2-3 hr) was used as a positive control. Cells were gently washed twice with PBS and incubated in live cell imaging solution (Invitrogen, A14291DJ). Fluorescence was measured at 581/590 nm (excitation/emission) for the reduced dye, and at 488/510 nm (excitation/emission) for the oxidized dye, while Hoechst was measured at 350/461 nm (excitation/emission) using the Infinite 200 PRO Tecan microplate reader (Tecan Inc., Switzerland). The green-to-red fluorescence intensity ratio was used to measure lipid peroxidation. The values were normalized to cells number in each well using Hoechst staining values. Confocal microscopy images of live cells stained with C11-BODIPY were acquired with the described excitation/emission wavelengths using the LSM800 (Zeiss), 40X oil lens and the ZEN Imaging Software. Lipid peroxidation was also estimated by the level of 4-HNE (4-hydroxynonenal) protein adducts as detected by anti-4-HNE antibody (AB5605, SIGMA) in WB analysis.

Mitochondrial lipid ROS

Lipid peroxidation within the mitochondria of live cells was detected by MitoPeDPP 452/470 fluorescence probe (M466, DOJINDO). Cells were plated in High-Content Imaging Glass Bottom 96-well Microplates (Cellvis) for ~24 hr and then treated with the indicated drugs. MitoPeDPP (0.1 mM in DMSO) was diluted 1:1000 in HBSS buffer containing 20 mM Hepes pH 7.4 and 1 mM Hoechst, and incubated with the cells for 25 min in cell culture incubator under dark. Cells were washed twice with HBSS/Hepes buffer, incubated in live cell imaging solution, and processed by confocal microscope LSM800 (Zeiss), 40X oil lens and the ZEN Imaging Software.

Annexin V staining and FACS analysis

Cells grown on coverslips in a 12-wells plate were treated with drugs as indicated and 24 hr later washed with PBS and then with Annexin V binding buffer (BB) (0.1 M Hepes, pH-7.4, 1.4 M NaCl and 25 mM $CaCl_2$). Cells were incubated with FITC-Annexin V (#640905, Biolegend; 1:1000 dilution in Annexin V BB) for 20 min in dark at room temperature. Cells were washed twice with Annexin V BB, fixed in 4% paraformaldehyde in Annexin V BB and processed by confocal microcopy [2].

For flow cytometry analysis, cells were treated with drugs for 24 hr, and then 1×10^6 viable cells (trypan blue negative) were collected, filtered through 70 µm Falcon cell strainers, wash with Annexin V BB and then stain with FITC-Annexin V as described above in 500 µl of Annexin V binding buffer. Samples were washed (5 min, 1200 rpm) with Annexin V BB, re-suspended in 300µl of Annexin V BB containing 1% BSA and analysed in BD Biosciences FACSAriaIII.

Iron measurement

Total cellular iron was measured by the iron colorimetric assay kit (#K390, BioVision). 1×10^6 cells were seeded in 10 cm plates, and 24 hr later were treated with the indicated drugs for additional of 24 hr, washed twice with cold PBS and lysed in 200 µl of lysis buffer containing 0.5% Triton X-100 and protease inhibitors (10 µg ml^{-1} leupeptin and 10 µg ml^{-1} aprotinin). Cell lysates were centrifuged at 14,000 rpm for 15 min and 100 µl was taken from supernatant for measuring total iron (Fe^{2+} and Fe^{3+}) according to the manufacturer's instructions, using a microplate reader (Infinite 200 PRO, Tecan) and absorbance at 590 nm. Results were normalized to total protein content in cell lysates and expressed fold of untreated control (DMSO).

Proteasomal activity assay

Proteasome activity was measured as previously described.[3] $2*10^5$ cells were seeded in 6-well plates, and 24 hr later treated with the indicated drugs for 24 hr. Cells were lysed in TNH buffer (20 mM HEPES, pH 7.5, 100 mM NaCl, 1mM EDTA, 1.5 mM $MgCl_2$ and 1% Triton x100), centrifuged at 13,500 rpm for 15 min at 4°C, and protein concentration of the supernatant was determined by Bradford assay (Bio-Rad, Hercules, CA). Cell lysates (30 µg total protein in 50 µl volume) were incubated with 100 µl of proteasome reporter substrate Suc-LLVY-AMC (Sigma S6510) (100 µM in 20 mM Tris pH 7.4, 100 mM NaCl) for 10-30 min at room temperature. Proteasomal degradation of the reporter released the fluorescent product 7-amino-4-methylcoumarin, which was measured at 340/440 nm (excitation/emission) using the Infinite 200 PRO Tecan. Values were normalized to total protein in cell lysates and presented as % inhibition relative to control.

Iron staining

Intracellular labile ferrous ion (Fe^{+2}) was detected by live cell imaging using FerroOrange (F374, DOJINDO) fluorescent probe (ex/em 561/570-620 nm). Cells were seeded in a 96 well glass bottom plate and treated with drugs as indicated, washed with PBS, and incubate with serum free RPMI media containing 1 µM of FerroOrange and 1 mM Hoechst for 25-30 minutes in dark condition. After washing with PBS, cells were incubated with live cell imaging solution, and either processed by confocal microscopy and/or were analyzed by Infinite 200 PRO Tecan microplate reader. Fluorescence was measured with Texas Red filter set (561/600 nm excitation/emission) for FerroOrange, and 4′,6-diamidino-2-phenylindole filter set (350/461 nm excitation/emission) for Hoechst staining. Red signal values were normalized to number of living cells in each well using Hoechst staining values.

β -hexaminidase activity assay.

$2*10^5$ cells were seeded in 6-well plates, and 24 hr later treated with the indicated drugs for 24 hr and then lysed with 100 μl of lysis buffer containing 0.1% Triton-X 100 and protease inhibitors (10 μg ml^{-1} leupeptin and 10 μg ml^{-1} aprotinin). Cell lysates were centrifuged at 15,000 rpm for 10 min and the supernatant was mixed 1:1 (vol./vol.) with 1mM of N-acetyl-β-glucosaminidase substrate, p-Nitrophenyl N-acetyl-β-D-glucosaminide (487052, SIGMA) in total volume of 30μl. Following 1 hr incubation at 37˚C, 220 μl of 0.1 M bicarbonate buffer (88975, SIGMA) was added to the reaction mix and absorbance was read at 400 nm using microplate reader (Infinite 200 PRO, Tecan). Results were normalized to total protein content in cell lysates and expressed as fold change of untreated control (DMSO).

Kinetic response of ferroptosis/apoptosis inducers

1×10^6 of MDA-MB-468 or HCC70 cells were seeded in 10 cm plates, and 24 hr later were treated with either IC_{50} doses of FIN drugs (1 μM Erastin, 200 nM FIN56, 180 nM RSL3) or with 25 nM staurosporine or 10 nM paclitaxel to stimulate apoptosis. Cells were harvested at 6, 12, 24, 32 hr post treatment and RNA was extracted for qPCR analysis.

Polar Metabolomics profiling

Extraction and analysis of lipids and polar metabolites was performed as previously described in [4] with some modifications: samples were extracted with 1 ml of a pre-cooled (−20˚C) homogenous methanol:methyl-tert-butyl-ether (MTBE) 1:3 (v/v) mixture, containing the following internal standards: 0.1 μg mL^{-1} of Phosphatidylcholine (17:0/17:0) (Avanti), 0.4 μg ml^{-1} of Phosphatidylethanolamine (17:0/17:0, 0.15 nmol/ml of Ceramide/Sphingoid Internal Standard Mixture II (Avanti, LM6005), 0.0267 μg ml^{-1} d5-TG Internal Standard Mixture I (Avanti, LM6000) and 0.1 μg ml^{-1} Palmitic acid-13C (Sigma, 605573). The tubes were vortexed and then sonicated for 30 min in ice-cold sonication bath (taken for a brief vortex every 10 min). Then, UPLC-grade water:methanol (3:1, v/v) solution (0.5 ml), containing the following internal standards: C13 and N15 labeled amino acids standard mix (Sigma, 767964), was added to the tubes followed by centrifugation. The upper, organic phase was transferred into 2 ml Eppendorf tube. The polar phase was re-extracted as described above, with 0.5 ml of MTBE. Both organic phases were combined and dried in speedvac and then stored at −80˚C until analysis. For analysis, the dried lipid extracts were re-suspended in 200 μl mobile phase B (see below) and centrifuged again at 13,000 rpm and 4˚C for 5 min. Lower, polar phase used for polar metabolite analysis was resuspended in 150 μl Methanol:DDW (50:50). LC-MS polar metabolite analysis was performed using Acquity I class UPLC System combined with mass spectrometer Q Exactive Plus Orbitrap™ (Thermo Fisher Scientific) as described in [5]. The data processing was done using TraceFinder (Thermo Fisher Scientific) as described in [5].

LC-MS for lipidomics analysis

Lipid extracts were analyzed using a Waters ACQUITY I class UPLC system coupled to a mass spectrometer (Thermo Exactive Plus Orbitrap) which was operated in switching positive and negative ionization mode. The analysis was performed using Acquity UPLC System combined with chromatographic conditions as described in [4a] with small alterations. Briefly, the chromatographic separation was performed on an ACQUITY UPLC BEH C8 column (2.1×100 mm, i.d., 1.7 μm) (Waters Corp., MA, USA). The mobile phase A consisted of DDW: Acetonitrile: Isopropanol 46:38:16 (v/v/v) with 1% 1 M NH_4Ac, 0.1% acetic acid. Mobile phase B composition is DDW: Acetonitrile: Isopropanol 1:69:30 (v/v/v) with 1% 1 M NH_4Ac, 0.1% acetic acid. The column was maintained at 40˚C and flow rate of mobile phase was 0.4 ml min^{-1}. Mobile phase A was run for 1 min at 100%, then it was gradually reduced to 25% at 12 min, following decrease to 0% at 16 min. Mobile phase B was run at 100% till 21 min, and mobile phase A was set to 100% at 21.5 min. Then, column was equilibrated at 100% A till 25 min. Orbitrap data was analyzed using LipidSearch™ software (Thermo Fisher Scientific). The validation of the putative identification of lipids was performed by

comparing to home-made library which contains lipids produced by various organisms and on the correlation between retention time (RT) and carbon chain length and degree of unsaturation.

Computational analysis of metabolite\lipidomics profile

Relative levels of polar compounds\lipids were normalized to the internal standards and the protein amount in the examined samples. They were further normalized to the total intensities of each sample. Fold changes and significance levels for differential metabolite were measured using Limma in R. Enrichment analysis was performed using Metaboanalyst.ca [6] using the "enrichment analysis" tool with KEGG as the reference dataset.

Glutamine tracing

1×10^6 MDA-MB-468 cells were seeded in a 10 cm plates and 24 hr later were treated with drugs combination in glutamine free RPMI media containing 2 mM $^{13}C_5$-gltumine (Cambridge Isotope laboratories, #CLM-1822-H-PK). After 24 hr plates were washed twice with a cold (4°C) PBS followed by incubation with intracellular metabolites extraction solution (300 µl per plate) containing; Methanol: Acetonitrile: Water at a ratio of 5:3:2 respectively for 10 min on a shaker at 4°C. Supernatants were collected, centrifuged at 16,000g for 10 min at 4°C and transferred to HPLC glass vials. Samples were kept at -80°C until processing in LC-MS. LC-MS analysis was conducted as described.[7] Briefly, Dionex Ultimate ultra-high-performance liquid chromatography (UPLC) system coupled to Orbitrap Q-Exactive mass spectrometer (Thermo Fisher Scientific) was used. Resolution was set to 35,000 at 200 mass to charge ratio ($m\ z^{-1}$) with electrospray ionization and polarity switching mode to enable both positive and negative ions across a mass range of 67–1000 $m\ z^{-1}$. UPLC setup consisted a ZIC-pHILIC column (SeQuant; 150 mm × 2.1 mm, 5 µm; Merck). 5 µl of cells or media extracts were injected using an auto sampler. Compounds were separated using a 15 min gradient, starting at 20% aqueous (20 mM ammonium carbonate adjusted to pH 9.2 with 0.1% of 25% ammonium hydroxide) and 80% organic (acetonitrile), terminated with 20% acetonitrile. Flow rate and column temperature were kept at 0.2 ml min^{-1} and 45 °C, respectively for a total run time of 27 min. All metabolites were detected using mass accuracy below 5 ppm. Thermo Xcalibur 4.4 was used for data acquisition. The peak areas of different metabolites were determined using Thermo TraceFinderTM 5.1 software. Metabolites were identified using the exact mass of the singly charged ion as well as the retention time of a matching standard, using an in-house library acquired by running commercial standards for all detected metabolites. Isotopologues analysis was done by verifying that the labeled signal retention time matches to the unlabeled signal retention time as well as using the labeled signal accurate mass.

Dataset analysis

To determine the clinical impact of the signatures designed, the MetaBric dataset of breast cancer patients was downloaded from cBioPotal.org. Further analysis was performed in R. ssGSEA method was used to score the enrichment of the GGS subsets in those patients, using the gsva() function (from the gsva package). The umap() function (with default parameters) was used to draw a UMAP projection of the TNBC patients, the kmeans() function to cluster each patient based on their UMAP coordinates, and a Kaplan-Meier plot was performed using the 'survival' package, and visualized using the 'survminer' package. Univariate and multivariate Cox regression was done using the coxph() function from the `survival` package, using the overall survival data included in the MetaBric dataset. Multivariate analysis was visualized using the forest_model() function in the forestmodel package. Similarly, the enrichment of the 'JB2 Up' subset was scored, using ssGSEA, in datasets of patients and PDX models, which contained chemotherapy response data.

Supplementary Text: Generation of gene signatures and biomarkers

This text describes the computational methods applied in this study, which were performed in R.

Generation of the Gradient Gene Set (GGS)

The analysis of JB2 and JB6 transcriptomic changes vs. DMSO and versus each other was used to establish four gene sets: 'JB2 Up', 'JB2 Down', 'JB6 Up', 'JB6 Down', each consists of genes with the highest or lowest fold change between JB2 and JB6 at 24 hr post treatment. The table below describes how the genes were selected for the four sets. Here, 'Significantly' means adjusted p-value (FDR) < 0.05, and 'FC' refers to the log2 fold-change. For example, the 'JB2 Up' set, consists of all genes which are significantly upregulated in JB2 vs. DMSO, **and not** significantly up-regulated in JB6 vs. DMSO; from all genes in the RNAseq, 529 genes pass this filter in MDA-MB-468, and 596 genes pass this filter in HCC70. Next, in each cell line, we took the 100 genes with the highest JB2 to JB6 fold change in that cell line (note: the cutoff for the gradient, 100, was chosen arbitrarily, to make this gene set relatively small). Hence, we had a list of 100 genes from MDA-MB-468 and 100 genes from HCC70, total 194 genes (6 genes overlap between the two cell lines). We then demanded that all genes in the final 'JB2 Up' set will be increased in JB2 vs. DMSO and JB2 vs. JB6 (even not significantly) in both cell lines. The final 'JB2 Up' set contains 75 genes, all of them are increased in JB2 vs. DMSO and vs. JB6 in both cell lines (significantly in at least one cell line, and in a trend in the other cell line). Similarly, the 'JB2 Down', 'JB6 Up' and 'JB6 Down' sets were built; all four sets together compose the Gradient Gene Set (GGS).

Supplementary text Table 1 – Flow chart for selecting genes for the 4 gradient gene set.

<table>
<tr><th>Group</th><th>JB2 Up</th><th>JB2 Down</th><th>JB6 Up</th><th>JB6 Down</th></tr>
<tr><td colspan="5">Starting with all genes in the RNAseq

Step 1: keep all genes that obey these rules in regard to their fold change in JB2 or JB6 vs. DMSO:</td></tr>
<tr><td>JB2 vs. DMSO</td><td>Significantly upregulated</td><td>Significantly downregulated</td><td>Not Significantly upregulated</td><td>Not Significantly downregulated</td></tr>
<tr><td>JB6 vs. DMSO</td><td>Not Significantly upregulated</td><td>Not Significantly downregulated</td><td>Significantly upregulated</td><td>Significantly downregulated</td></tr>
<tr><td colspan="5">Step 2: rank the genes by JB2 vs. JB6 fold change and keep the top 100 genes with:</td></tr>
<tr><td>JB2 vs. JB6</td><td>the highest JB2 vs. JB6 fold change</td><td>the lowest JB2 vs. JB6 fold change</td><td>the lowest JB2 vs. JB6 fold change</td><td>the highest JB2 vs. JB6 fold change</td></tr>
<tr><td colspan="5">Step 3: combine all genes that follow the above rules in either one of the cell lines (MDA-MB-468 or HCC70), and in the other cell lines obey the rules below:
(i.e, to be in the GGS the gradient needs to be significant in one cell line and in a trend in the other)</td></tr>
<tr><td>Log2 FC of JB2 vs. DMSO</td><td>> 0</td><td>< 0</td><td>-</td><td>-</td></tr>
<tr><td>Log2 FC of JB6 vs. DMSO</td><td>-</td><td>-</td><td>> 0</td><td>< 0</td></tr>
<tr><td>(Log2 FC of JB2 vs. DMSO) – (Log2 FC of JB6 vs. DMSO)</td><td>> 0</td><td>< 0</td><td>< 0</td><td>> 0</td></tr>
<tr><td colspan="5">Final number of genes in each group (total = 306 genes):</td></tr>
<tr><td>N =</td><td>75</td><td>71</td><td>87</td><td>73</td></tr>
</table>

Public datasets of ferroptosis and apoptosis inducers

To assess the classification accuracy of the GGS between the ferroptosis and apoptosis landscapes, we collected published transcriptomic data (RNAseq) of 19 ferroptosis and 26 apoptosis inducers from different cancer cell lines (Table S1; FIN datasets F1-F19, AIN datasets A1-A26). We only included data with repeats, for which statistical significance can be calculated. The transcriptomic data were downloaded from the GEO repository, either manually or by using the getGEO() function from the GEOquery library in R. In most cases, we downloaded the count matrix, filtered low expressing genes, normalized to library size and calculated log CPM as we did for our RNAseq, using the edgeR package in R. In a few cases, the normalized count data was already available in the GEO record. Since we could only find 19 FIN datasets (and many more AINs datasets), we also generated 5 FIN datasets for validation by performing RNAseq on basal TNBC cell lines (MDA-MB-468, HCC70, HCC38) treated with erastin or RSL3. In total, we used 50 datasets (19 FINs, 26 AINs, 5 FINs we created for validation).

Next, we used Limma in R to calculate several gene-level statistics in each dataset: The log2 fold change for every gene between the inducer and its vehicle control, and the moderated t-statistics – the ratio of the log2 fold change and its moderated standard error – from which the p-values for each gene were derived. The 50 datasets were obtained by different experimental settings and/or different research teams and in some cases using different RNA-sequencing methodology. Most importantly, the 50 datasets contain different numbers of total genes, which requires normalization. Therefore, we ranked the genes based on their signed t-statistic, which was previously used for enrichment methods including gene set enrichment analysis.[8] In each dataset, the genes were ranked by their signed t-statistics, and then the ranks were scaled from 0 (for the most downregulated gene by the inducer vs. its control) to 1 (for the most upregulated gene by the inducer vs. the control). Ranking by the t-statistics takes into account both the actual fold change in gene expression and the standard error of the repeats. Ranking by t-statistics also has the advantage of reducing the effect of outliers in the data; however, it might reduce the distance between clusters since the ranking limits the dynamic range of gene expression to a scale from 0 to 1.

UMAP visualization of ferroptosis to apoptosis datasets segregation by the gradient genes

To visualize the classification capacity of the GGS (n = 306 genes) on a 2D plane, the UMAP dimension reduction method was used. UMAP is a non-linear dimension reduction method, superior to other dimension reduction methods such as PCA as it preserves local neighborhoods and the global structure of the data,[9] and was recently shown to effectively cluster bulk RNAseq data.

First, we combined all the ranked t-statistics of the 50 FINs and AINs datasets into a single matrix and filtered the genes to include only the genes of the Gradient gene set (GGS). Since the datasets are from different resources, missing data could raise an issue, as certain genes might be included in only one or two datasets, and thus are candidates for removal. On the other hand, important genes for classification might be missing only in a few datasets, possibly due to the low count filter in the count matrix analysis. Those genes are candidate for missing data imputation. We decided to remove all genes which have more than 12 missing data among the 50 datasets. A gene with 12 or less missing data was imputed by using the median expression of that gene in the other ferroptosis or apoptosis datasets.

The UMAP projection was built using the umap() function from the UMAP package in R, using the default parameters. A random seed was set for reproducing the results. This generated a 2D plane with the 19 FINs, 26 AINs, and 5 validation sets (Figure 2C). To quantify the classification accuracy of the 50 FINs and AINs datasets into ferroptosis and apoptosis, we built a k-nearest neighbors (kNN) model using the tidymodels package in R. The predictors for the model were the ranked t-statistic levels of the GGS genes (dealing with missing data as mentioned above). The model was trained for the number of neighbors (k=5-15), using 5-fold cross validation. ROC-AUC (area under the receiver operating characteristic curve) was used as performance metric. The value of k that gave the best AUC was chosen.

To emphasis the ferroptosis and apoptosis region of the 2D plane, we created contour plot of ferroptosis probabilities on top of the UMAP projection using another kNN model, which was built using the UMAP x-

and y- coordinates of the datasets as predictors. To that end, a grid of ~90,000 equally spaced points were positioned in the plane, and this kNN model was used to predict the ferroptosis probability in each such point. This procedure created the areas of probabilities, from 100% ferroptosis (green) to 100% apoptosis (blue). Importantly, the AUC reported is derived from the kNN model using the GGS genes, and not the UMAP coordinates, and is therefore not affected by the visualization method or the UMAP parameters.

Generation of the datasets-derived biomarkers, a biased predictor

Biomarkers with the most extreme log2 fold changes in the 19 FINs and the 26 AINs public datasets should be the best biomarkers to distinguish between the two cell death pathways. However, such biomarkers are biased to the 45 datasets. To reduce this bias, we adapted a method from [10], in which 12 FINs datasets were randomly selected out of the total 19 (selection with replacement, meaning a dataset can be selected more than once). The mean fold change of each gene in those 12 datasets was calculated. Similarly, this was done for 12 randomly selected AINs datasets (out of total 26, with replacement). We then calculated, for each gene *i*, this difference:

$$d_i = (mean\ log_2(fold\ change)\ in\ the\ 12\ FINs\ datasets) - (mean\ log_2(fold\ change)\ in\ the\ 12\ AINs\ datasets)$$

We selected the 150 genes with the highest d_i, representing the genes most upregulated in the FINs datasets vs. the AINs, and 150 genes with the lowest d_i, representing the genes most upregulated in the AINs datasets vs. the FINs. This process was repeated 1000 times, each time randomly picking 12 FINs and 12 AINs datasets and calculating the d_i for each gene.

To build the final dataset-derived biomarkers set, each gene *j* received the following score, based on the 1000 iterations performed above. Let n be the iteration number ($n = \{1,2, \ldots ,1000\}$), and let $f_j(n)$ be equal to 1 if the gene *j* is one of the 150 genes with the *highest* d_i in the nth iteration (thus being upregulated in ferroptosis), or 0 otherwise. Similarly, let $a_j(n)$ be equal to 1 if the gene *j* is one of the 150 genes with the *lowest* d_i in the nth iteration (thus being upregulated in apoptosis), or 0 otherwise. The score for gene *j* will be:

$$score_j = \left(\sum_{n=1}^{1000} f_j(n)\right) - \left(\sum_{n=1}^{1000} a_j(n)\right)$$

For example, DDIT4 appears in 936 out of the 1000 iterations as part of the 150 genes with the highest d_i (favoring ferroptosis), and never among the 150 genes with the lowest d_i, and therefore its score would be 936 (the highest of all genes). Consequently, this gene would be one of the best markers for ferroptosis vs. apoptosis. Figure S2D presents the scores of all genes. The genes are ranked by their score from highest (most favoring ferroptosis) to the lowest (most favoring apoptosis). For biomarkers, we picked the 15 highest scoring genes (Figure 2D).

Generating the gradient-derived biomarkers set, an unbiased predictor set of ferroptosis

The dataset-derived biomarkers, described above, consists of most differentially expressed genes between the 19 FINs and the 26 AINs datasets. However, this set is biased to those datasets, and thus might have a lower prediction capacity for new datasets. Nevertheless, the diversity of the ferroptosis datasets (different settings/inducers) might mitigate this issue. To generate an unbiased set of ferroptosis vs. apoptosis biomarkers, we aimed to use only the RNAseq we generated for JB2 and JB6 vs. DMSO and vs. each other, and than calculate the classification accuracy using the public FINs and AINs datasets. Although the GGS could classify the FINs and AINs datasets with high accuracy (Figure 2C), the UMAP projection was not appropriate to define the best ferroptosis biomarkers (i.e., the genes which are upregulated in ferroptosis vs. apoptosis specifically).

We noticed that many of the datasets-derived biomarkers are increasing in JB2 vs. DMSO and vs. JB6, as expected, 24 hr post-treatment, while tending to decrease in JB2 vs. JB6 at 6 hr post-treatment. Therefore, for the unbiased approach, we took one set containing all genes with a significant (FDR < 0.05) fold change increase in JB2 vs. JB6, at the 24 hr timepoint (in total, 332 genes), and a second set containing all genes with a significant fold change decrease in JB2 vs. JB6 at the 6 hr timepoint (in total, 436 genes). Those two sets intersect to give 26 genes which 'inverse' their JB2-to-JB6 gradient from 6 hr to 24 hr post-treatment. The 26 genes were ranked according to their fold change between JB2 and JB6 in 24 hr, with the highest fold change in JB2 vs. JB6 ranked 1st.

Evaluating the classification accuracy of the biomarkers

To validate the biomarkers using the public FINs and AINs datasets, we used single-sample gene set enrichment analysis (ssGSEA), using the gsva() function in the GSVA package in R, with the method parameter set to "ssgsea". We scored the enrichment of the datasets-derived biomarkers (top 15-400 genes), the gradient-derived biomarkers (top 15-26 genes), a representative ferroptosis signature (erastin in HT1080 set of 33 genes, marked F1 in our datasets lists), and a public apoptosis signature ("hallmark apoptosis" set, 154 genes). We than calculated 3 statistics (presented in Figure 2D):

(1) To measure the classification accuracy, we calculated the ROC-AUC between the scores of the 26 AINs datasets, and those of the 19 FINs public datasets plus our 5 FIN RNAseq datasets. The AUCs were calculated using the auc() function from the pROC package. P-value of the AUCs was determined as follows: we generated 1000 gene sets, each compose of 20 randomly selected genes. We than performed ssGSEA to score the enrichment of these random sets in the FINs and AINs datasets, and thereafter generated 1000 AUCs. We compared the AUC for the biomarkers genes to the distribution of these random 1000 AUCs to calculate the p-values.

(2) Direct comparison of the ssGSEA scores in the 24 FINs (19 public plus our 5) vs. the 26 AINs datasets was done using two sample t-test.

(3) To show that the FINs datasets have high ssGSEA score, suggesting that these genes are indeed biomarkers up-regulated by the FINs, we performed one-sample t-test of the ssGSEA scores vs. the baseline, 0 (which indicates no enrichment). In Figure 2D we marked only the non-significant groups (those whose enrichment score is not significantly different from 0) with "#".

Constructing the transcriptomic landscape figure

The apoptosis-ferroptosis plane, shown in Figure 1L, was used to identify the global transcriptomic landscape of the combinations (JB2, JB3 and JB6). To generate it, we used 3 public datasets of triple negative breast cancer (TNBC): **(1)** For apoptosis, 5FU-treated MDA-MB-231 cells (GSE124715, dataset #A4 in the list in supplemental Table S1). **(2)** For ferroptosis, ML162 in MDA-MB-231 (GSE162069, representing GPX4 inhibitors, or class II FINs), and erastin in HCC38 (GSE154425, representing system Xc^- inhibitors, or class I FINs), datasets #F16 and #F18 in the list in supplemental Table S1.

The three datasets were normalized by ranking the t-statistics as above. For the two ferroptosis datasets, we combined both datasets (representing the two most popular classes of FIN inducers), removed genes with missing values, and calculated for each gene the mean ranked t-statistics in both ferroptosis datasets. Next, for each gene *i* we calculated the following score:

$$score_i = (mean\ ranked\ t\ statistics\ in\ ferroptosis) - (ranked\ t\ statistics\ in\ apoptosis)$$

The 150 genes with the highest scores compose the ferroptosis TNBC signature, and the 150 genes with the lowest scores compose the apoptosis TNBC signature. We then used gene set variation analysis (GSVA) to measure the enrichment of these two signatures in the public datasets used to generate the signatures (A4, F16 and F18), as well as in our RNAseq (JB2, JB3, JB6 in MDA-MB-468 and HCC70). We plotted those GSVA score corresponding to the ferroptosis signature in the x-axis and the apoptosis signature in the y-axis.

References for Supplementary Text

[1] S. Kim, A. Kedan, M. Marom, N. Gavert, O. Keinan, M. Selitrennik, O. Laufman, S. Lev, *EMBO Rep* **2013**, *14* (10), 891.

[2] a) A. K. Muller, U. A. Kohler, S. Trzebanski, Y. Vinik, H. M. Raj, J. A. Girault, N. Ben-Chetrit, A. Maraver, S. Jung, S. Lev, *Adv Sci (Weinh)* **2022**, *9* (9), e2105696, https://doi.org/10.1002/advs.202105696; b) N. Verma, O. Keinan, M. Selitrennik, T. Karn, M. Filipits, S. Lev, *Nature communications* **2015**, *6*, 6064, https://doi.org/10.1038/ncomms7064.

[3] E. Isakov, A. Stanhill, *J Biol Chem* **2011**, *286* (35), 30274, https://doi.org/10.1074/jbc.M111.240309.

[4] a) S. Malitsky, C. Ziv, S. Rosenwasser, S. Zheng, D. Schatz, Z. Porat, S. Ben-Dor, A. Aharoni, A. Vardi, *New Phytol* **2016**, *210* (1), 88, https://doi.org/10.1111/nph.13852; b) L. Zheng, S. Cardaci, L. Jerby, E. D. MacKenzie, M. Sciacovelli, T. I. Johnson, E. Gaude, A. King, J. D. Leach, R. Edrada-Ebel, A. Hedley, N. A. Morrice, G. Kalna, K. Blyth, E. Ruppin, C. Frezza, E. Gottlieb, *Nature communications* **2015**, *6*, 6001, https://doi.org/10.1038/ncomms7001.

[5] P. Vyas, S. Malitsky, M. Itkin, D. S. Tawfik, *J Am Chem Soc* **2023**, https://doi.org/10.1021/jacs.2c08636.

[6] Z. Pang, J. Chong, G. Zhou, D. A. de Lima Morais, L. Chang, M. Barrette, C. Gauthier, P. E. Jacques, S. Li, J. Xia, *Nucleic Acids Res* **2021**, *49* (W1), W388, https://doi.org/10.1093/nar/gkab382.

[7] G. M. Mackay, L. Zheng, N. J. van den Broek, E. Gottlieb, *Methods Enzymol* **2015**, *561*, 171, https://doi.org/10.1016/bs.mie.2015.05.016.

[8] G. Korotkevich, V. Sukhov, N. Budin, B. Shpak, M. N. Artyomov, A. Sergushichev, *bioRxiv* **2021**, 060012, https://doi.org/10.1101/060012.

[9] Y. Yang, H. Sun, Y. Zhang, T. Zhang, J. Gong, Y. Wei, Y. G. Duan, M. Shu, Y. Yang, D. Wu, D. Yu, *Cell Rep* **2021**, *36* (4), 109442, https://doi.org/10.1016/j.celrep.2021.109442.

[10] S. Caruso, A. L. Calatayud, J. Pilet, T. La Bella, S. Rekik, S. Imbeaud, E. Letouze, L. Meunier, Q. Bayard, N. Rohr-Udilova, C. Peneau, B. Grasl-Kraupp, L. de Koning, B. Ouine, P. Bioulac-Sage, G. Couchy, J. Calderaro, J. C. Nault, J. Zucman-Rossi, S. Rebouissou, *Gastroenterology* **2019**, *157* (3), 760, https://doi.org/10.1053/j.gastro.2019.05.001.

Supplemental Figures

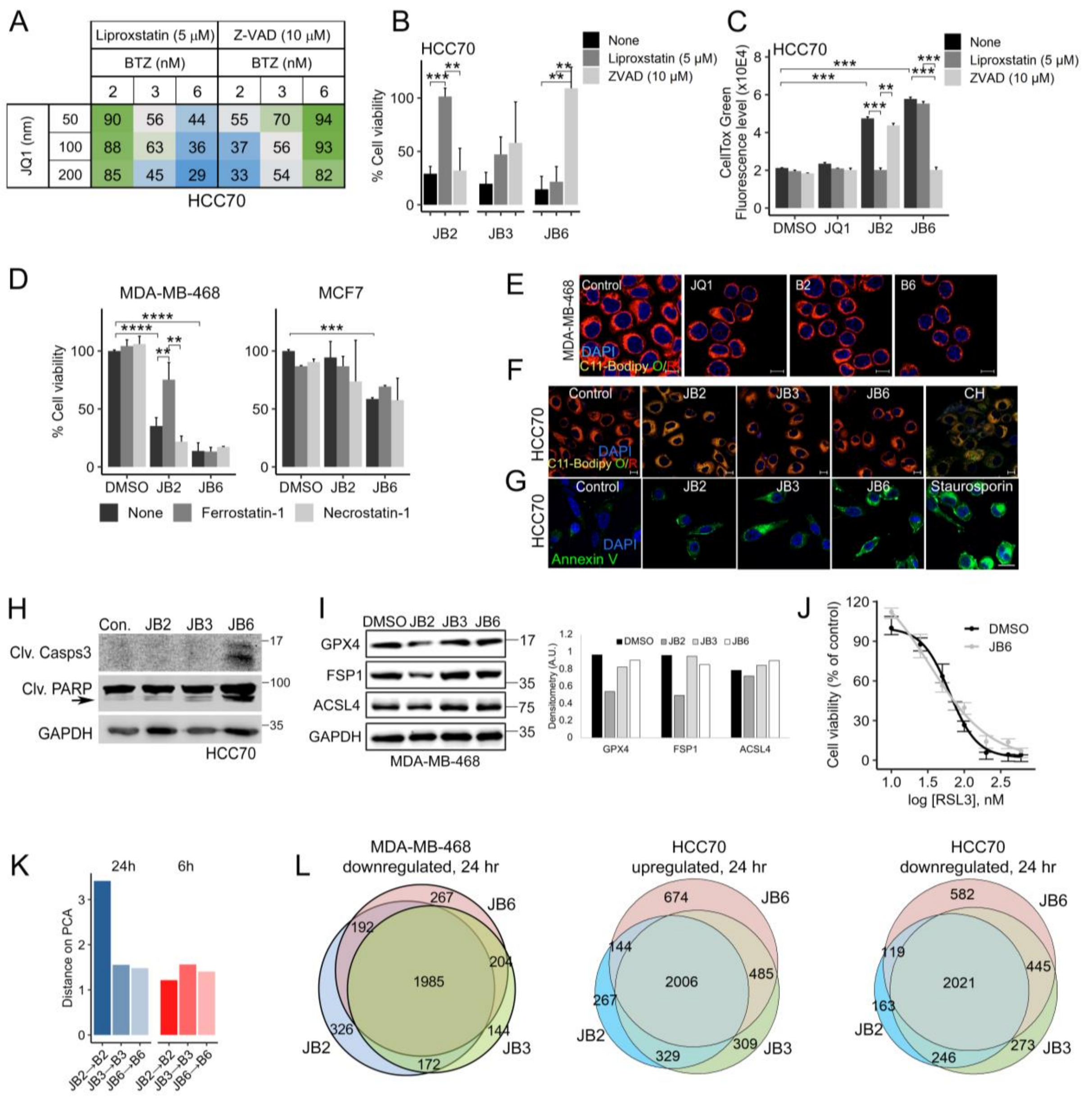

Figure S1: Ferroptosis-to-apoptosis transition by JQ1 and bortezomib combination

(A-C) HCC70 basal-like breast cancer cells were treated with either the indicated concentrations of JQ1 and BTZ (**A**) or 100 nM JQ1 together with 2 nM (JB2), 3 nM (JB3) or 6 nM (JB6) BTZ (**B**) in the absence or presence of liproxstatin-1 (5 μM) or z-VAD-FMK (10 μM). Cell viability was measured 72 hr later by MTT (**A, B**). Cell death was measured at the same time point by CellTox green (**C**). Percent (%) of cell viability (**A, B**) was calculated compared to control, and mean values ± SD of 3 (**B**) or 2 (**C**) independent experiments are shown, or a representative result (in A). P-values were measured by t-test.

(D) MDA-MB-468 and MCF7 cells were treated with JB2 or JB6 in the absence or presence of ferrostatin-1 (5 μM) or Necrostatin-1 (20 μM). Cell viability was measured 72 hr later by MTT. Percent (%) of cell viability was calculated compared to control and mean values ± SD of 2 repeats are shown. P-values were measured by t-test.

(E) Representative confocal images of MDA-MB-468 cells treated with JQ1, BTZ 2nM or BTZ 6nM for 24hr and stained with C11-BODIPY is described in Methods. Scale bar, 10 μm

(F, G) Representative confocal images of HCC70 cells treated with the drug combinations and stained with C11-BODIPY (F) or Annexin V (G). Cumene hydroperoxide (CH) 70μM for 2-3 hrs (F) or staurosporine (20 nM) for 24 hr (G) were used as positive controls. Scale bar, 10 μm (F), 20 μm (G).

(H) Western blot analysis for cleaved caspase-3 and PARP in HCC70 cells in response to drugs treatment for 24 hr.

(I) Western blot analysis for ferroptosis-related proteins in MDA-MB-468 24hr post drugs treatments.

(J) Similar confluency of control (DMSO) or JB6-pre-trearted MDA-MB-468 cells for 48 hr were incubated with the indicated concentrations of RSL3. Cell viability was measured 72 hours later by MTT. Data were calculated as percent of control. Mean values ± SD of two repeats are shown.

(K) Distances on the PCA plot (Figure 1K) between the JB2, JB3 and JB6 combinations to their respective single BTZ treatment (B2, B3 and B6, respectively). Distances are measured by Euclidean distance on the two major principal components (PC1 and PC2), taking the percentage of variance explained by the two PCs into account.

(L) Venn diagrams of significantly downregulated genes (FDR < 0.05) in MDA-MB-468, or up and down regulated in HCC70, in response to JB2, JB3 and JB6 vs. DMSO.

**p-value < 0.01, ***p-value < 0.001, ****p-value < $1x10^{-4}$

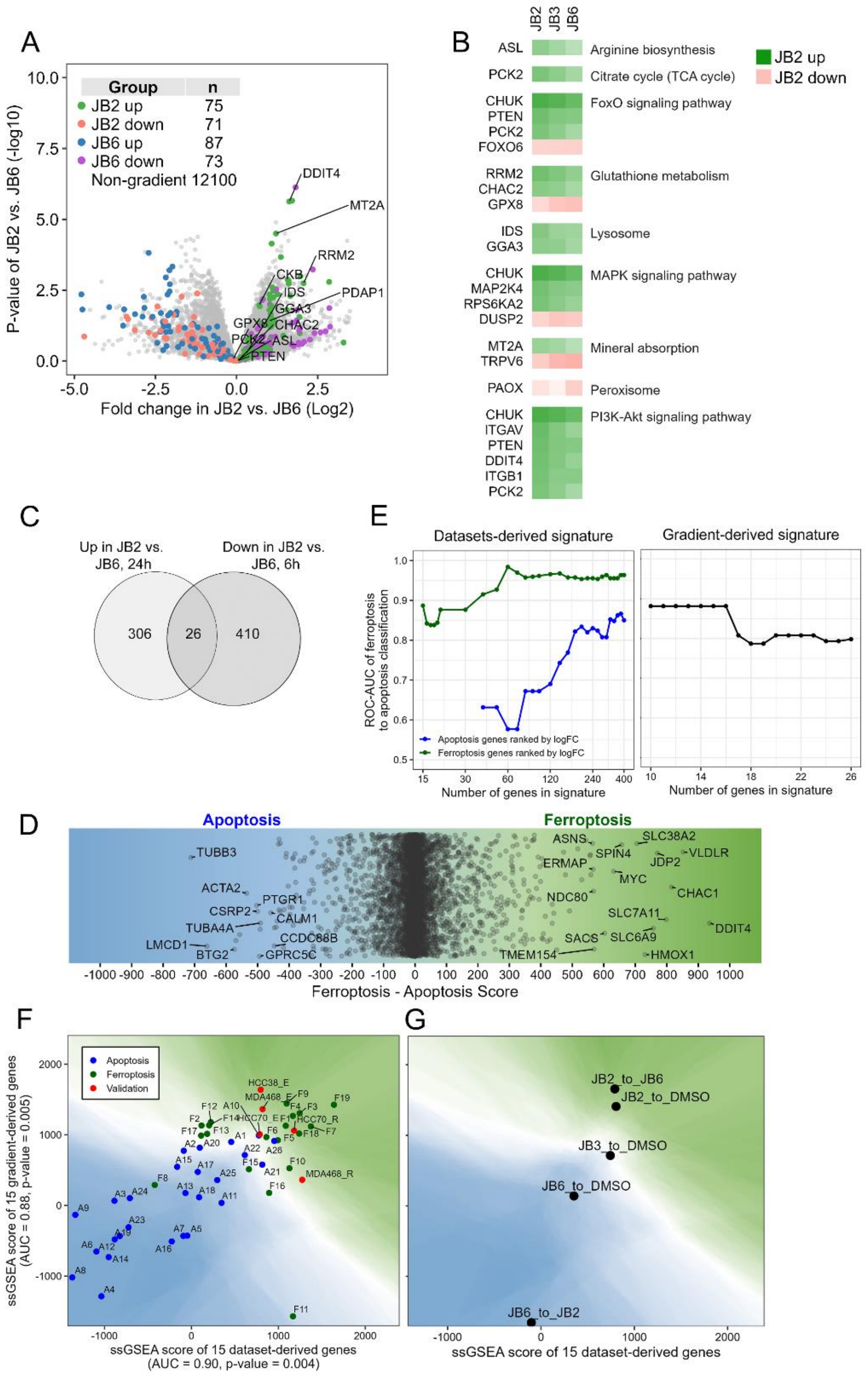

Figure S2: Development and validation of GGS and selective ferroptosis vs. apoptosis biomarkers.

(A) Volcano plot highlighting the four subsets of the gradient gene set. Data is based on RNAseq performed on HCC70.

(B) The GGS contains ferroptosis related genes. Color scale correlates to the log2 fold change in gene expression vs. DMSO.

(C) The 'Gradient-derived' biomarkers (GDB) are the 26 genes found in the intersection of two groups of genes: upregulated genes in JB2 vs. JB6 at 24 hr, and downregulated genes in JB2 vs. JB6 at 6 hr.

(D) The 'Dataset-derived' biomarkers (DDB) were developed by scoring each gene (x-axis) based on its expression in the 19 FINs and 26 AINs datasets. Genes with the highest scores are the best FINs vs. AINs biomarkers.

(E) FINs vs. AINs classification accuracy, given by ROC-AUCs, were determined based on the 19 FINs and 26 AINs datasets, for different GDB or DDB signatures sizes. For DDB, genes were sorted based on their scores (as shown in D). For the GDB, the genes were sorted based on the expression in JB2 vs. JB6 at 24 hr in MDA-MB-468.

(F-G) Enrichment scores for the top 15 DDB genes and GDB genes, were calculated by ssGSEA in the 19 public FIN datasets (green dots), 26 AINs datasets (blue dots), 5 FIN validation sets (red dots), and the JB2-3-6 combinations (black dots in G). Contour plots were created using k-nearest neighbors method.

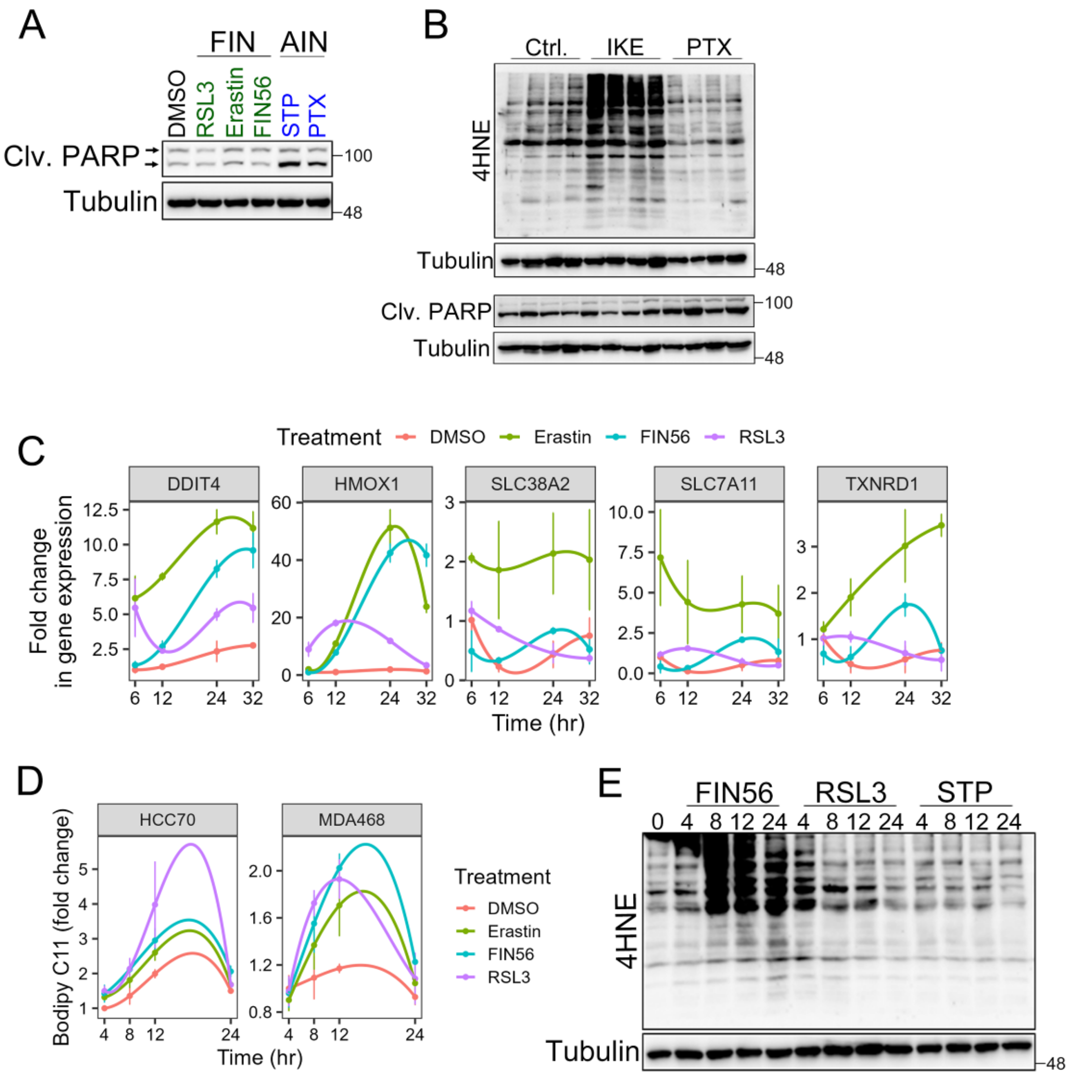

Figure S3: In-vitro and in-vivo validation of ferroptosis vs apoptosis biomarkers

(A) Cleaved PARP, a hallmark of apoptosis, is shown by WB for the AINs used in the in vitro validation of MDA-MB-468 cells, staurosporine (STP) and paclitaxel (PTX). Inducers were applied at IC_{50} concentrations for 24 hr.

(B) In vivo induction of ferroptosis/apoptosis using IKE/PTX. 2×10^6 MDA-MB-468 cells were implanted bilaterally into mammary fat pad of female nude mice. When tumors reached 50 mm^3, mice were randomized into three groups; IKE, PTX and control (Ctrl). Tumor lysates (n=4/group) from mice treated with drugs for 96 hr were analyzed by WB for lipid peroxidation using 4HNE antibody and cleaved PARP for apoptosis. Tubulin was used as a loading control.

(C-E) MDA-MB-468 were treated with the FINs (IC_{50}) for the indicated time periods. Expression of selected biomarkers was determined by qRT-PCR (**C**)**.** Onset of lipid peroxidation was quantified by BODIPY C-11 (**D**)**,** and is also shown by WB using anti- 4HNE antibody (**E**). Numbers above the gel indicate time post treatment in hrs.

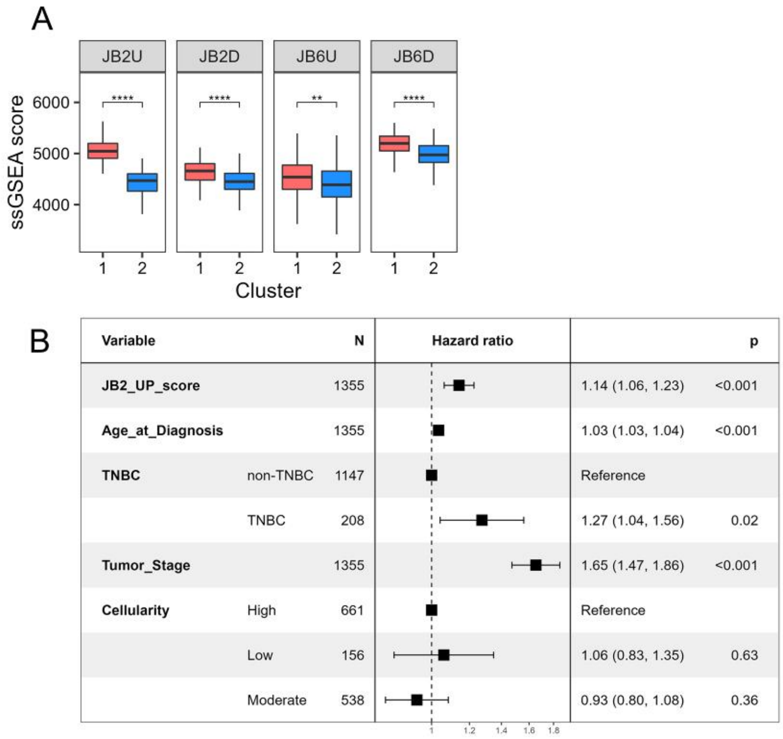

Figure S4: The `JB2 Up` set has a prognostic value

(A) The GGS subsets enrichment scores, calculated by ssGSEA, for patients in both clusters depicted in Figure 3A.
(B) Multivariate Cox regression using the 'JB2 Up' enrichment scores for MetaBric patients (n = 1904), together with other clinical factors available in the MetaBric dataset.

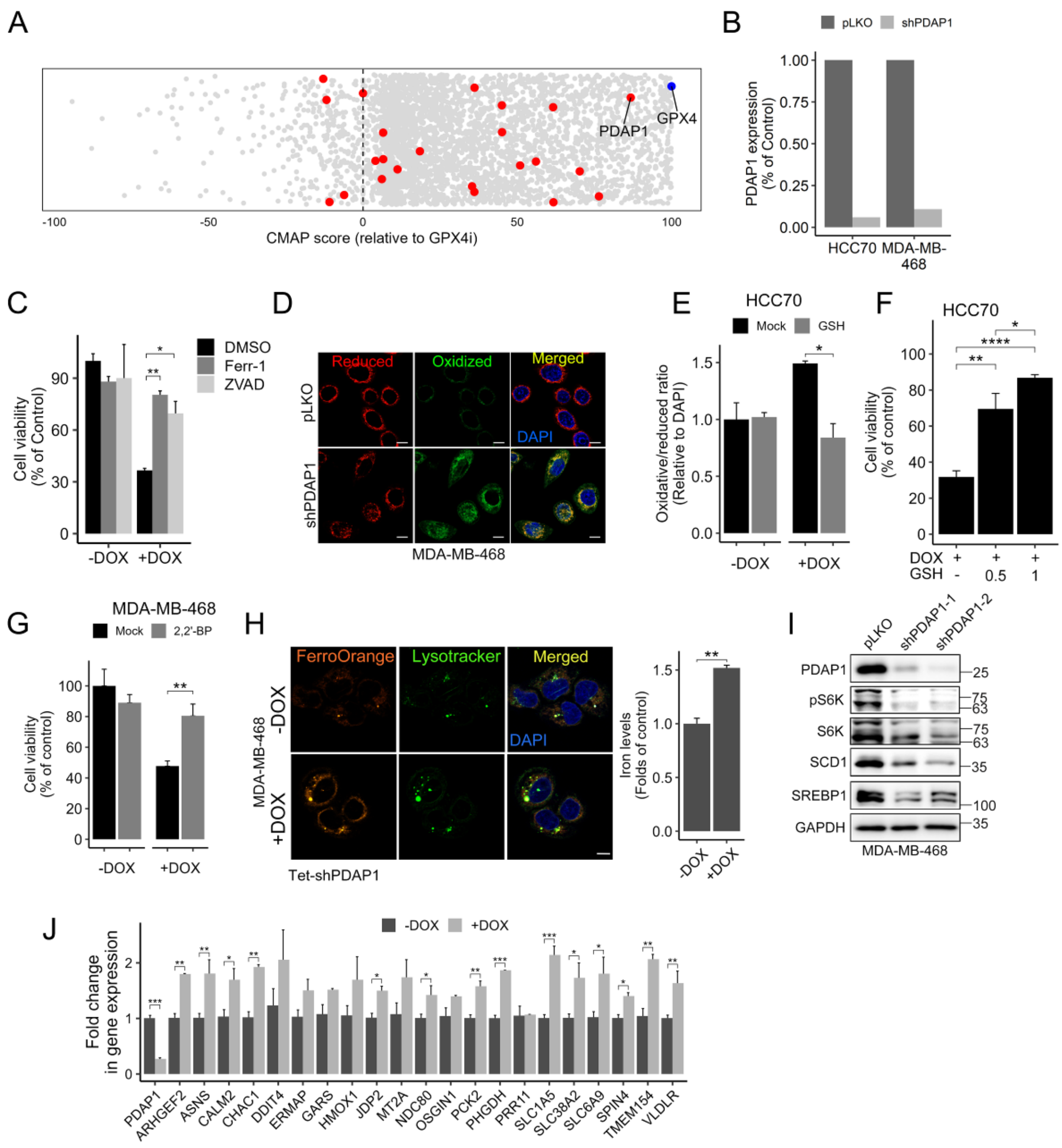

Figure S5: PDAP1 negatively regulates ferroptosis

(A) Similarity between the transcriptomic effect of GPX4 inhibition to ~3800 other genes, measured using the Connectivity Map Project (Broad). The score is on a scale from 100 (most similar perturbations to GPX4) to -100. Red dots indicate the 'JB2 Up' genes, which were included in this analysis.

(B) PDAP1 knockdown was validated by qRT-PCR in MDA-MB-468 and HCC70.

(C) MDA-MB-468 cells expressing the doxycycline (DOX) inducible PDAP1 knockdown (KD) were treated with either 1 μg ml^{-1} of DOX alone or together with 5μM ferrostatin-1 or 10μM ZVAD for 96 hrs. Cell viability was measured by MTT. Shown are means ± SD of two repeats. P-values were measured by t-test.

(D) Lipid peroxidation in MDA-MB-468 with PDAP1 knockdown was measured by BODIPY C-11 staining. Representative confocal images are shown. Scale bar 10μM.

(E-G) HCC70 (E,F) and MDA-MB-468 (G) cells expressing the inducible PDAP1 KD were treated with either 1 μg ml^{-1} of DOX alone or together with GSH (0.5-1mM) or 2,2'-BP (5 μM) for 48 hr (in E) and 96 hour (in F and G). The ratio of oxidized/reduced fluorescence of BODIPY-C11 signals was normalized to DAPI and is

shown in E. Influence on cell viability was measured by MTT assay and is presented as % of control (-DOX) (F). Shown are means ± SD of two (E), or 3 (F,G) repeats. P-values were measured by t-test.

(H) Lysosomal iron was assessed by co-staining of FerroOrange with lysotracker in control and PDAP1 depleted MDA-MB-468 cells (± DOX). Left: representative images. Right: FerroOrange signal was quantified and mean values ± SD of 2 independent repeats are shown. p-values were measured by t-test.
(I) Western blot analysis of the mTOR-SREBP axis done in MDA-MB-468 with PDAP1 knockdown. GAPDH was used as a loading control.
(J) In vivo validation of biomarkers following inducible KD of PDAP1. Expression level of PDAP1, and 21 selective ferroptosis biomarkers (Figure 2F), examined by qRT-PCR using RNA taken from the excised tumors (shown in Figure 4T).
*p-value < 0.05, **p-value < 0.01, ***p-value < 0.001

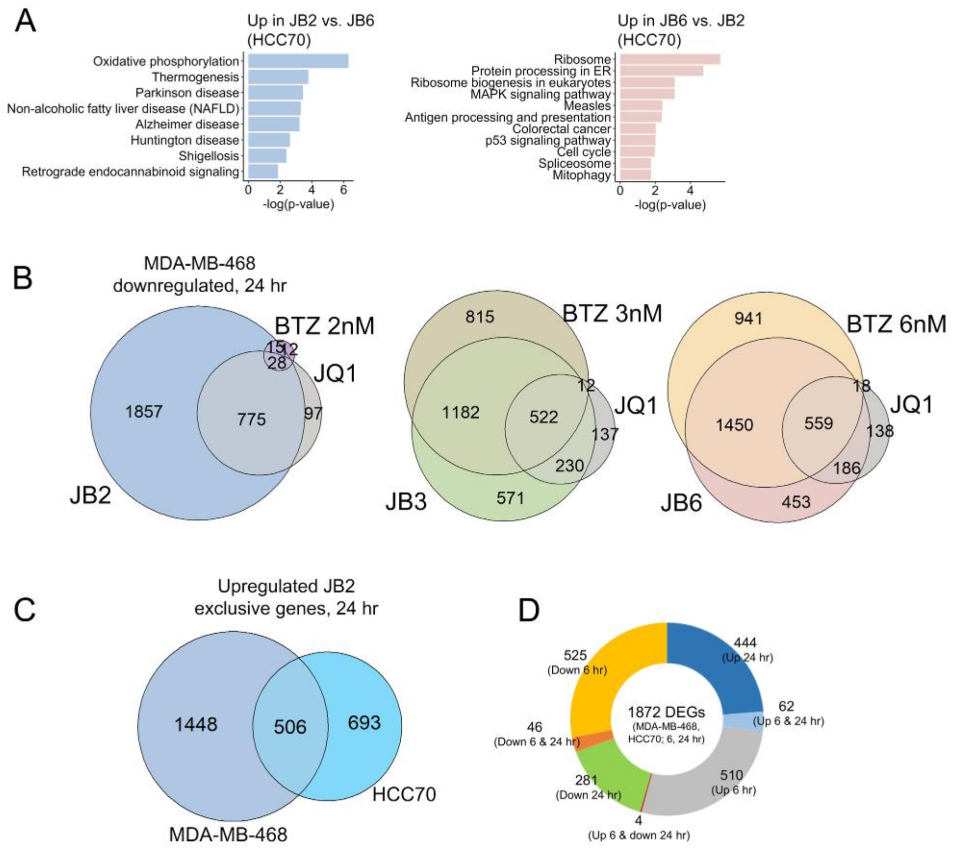

Figure S6: Transcriptomic analysis of JB2, JB3 and JB6 treatments

(A) KEGG pathways enrichment for genes upregulated in JB2 vs. JB6 (left) or JB6 vs. JB2 (right) in HCC70. **(B)** Venn diagram of significantly downregulated genes in JB2, JB3 or JB6 and their single components vs. DMSO, in MDA-MB-468.

(C) Venn diagram comparing the upregulated JB2 exclusive genes (genes upregulated by JB2, but not by JQ1 and B2) in both MDA-MB-468 and HCC70 cell lines.

(D) Pie chart depicting the number of JB2 exclusive genes, up- or down-regulated after 6 or 24 hours.

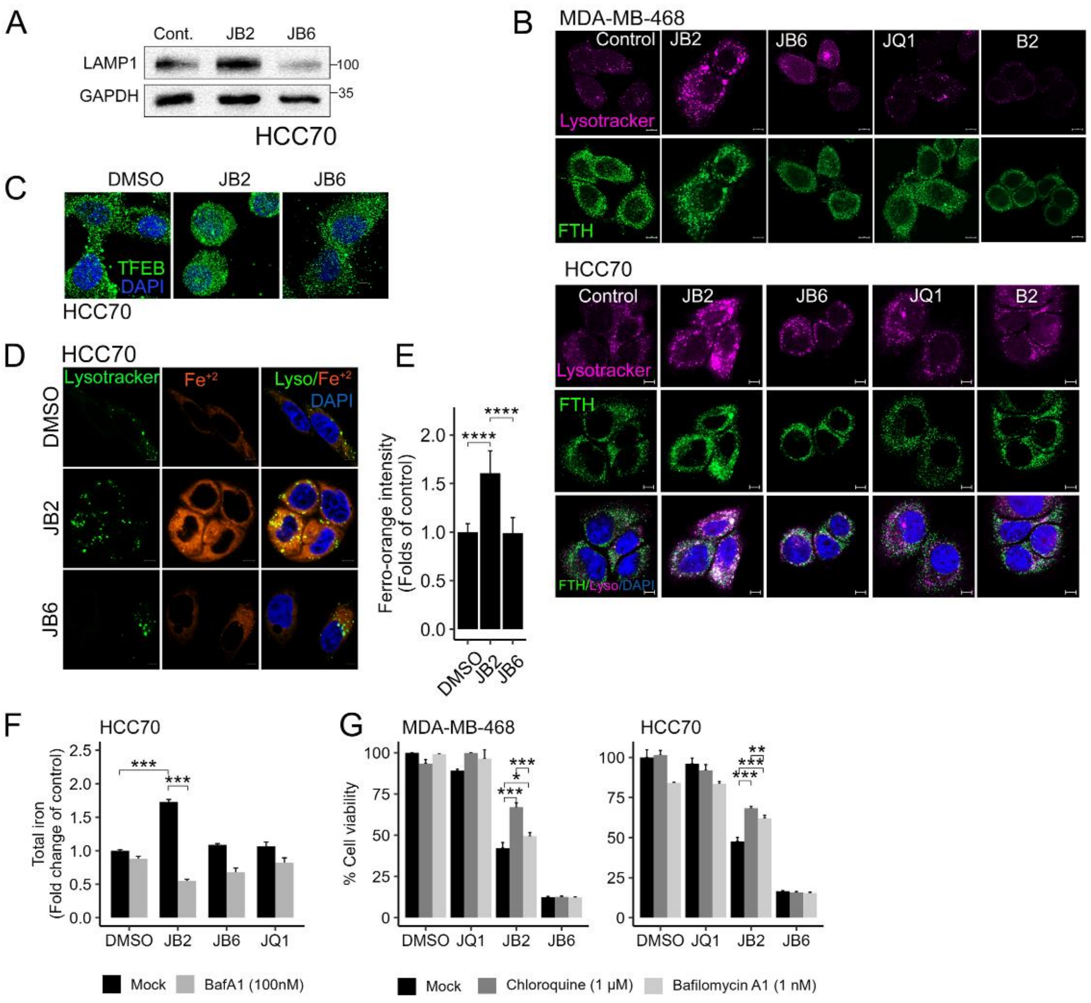

Figure S7: Lysosome associated activities in JB2-treated HCC70 cells.

(A, C) HCC70 cells were treated with the indicated drug combinations and 24 hr later the level of LAMP1 (**A**) or TFEB localization (**C**) was assessed by WB or IF analysis, respectively. Scale bar, 5 µm.

(B) Enhanced lysotracker staining and/or its colocalization with ferritin in JB2-treatead MDA-MB-468 and HCC70 cells. Representative confocal images are shown. Scale bar, 5 µm. Note: the MDA-MB-468 images are split-channels images of the merged images shown in Figure. 6K (main manuscript).

(D) Live cell imaging showing colocalization of lysotracker green with labile iron Fe^{+2} (FerroOrange), 24 hr post drugs treatment in HCC70, Scale bar, 10 µm.

(E) Quantification of the FerroOrange from D. Shown are means ± SD of 4 repeats; p-values were measured by t-test.

(F) HCC70 cells were treated with the indicated drugs in the presence or absence of bafilomycin A1 (Baf-A1) (100 nM) for 24 hr. Total iron measurement is shown as fold of control. Experiment was repeated twice in duplicates. Shown is a representative experiment. P-values are evaluated by t-test.

(G) MDA-MB-468 and HCC70 cells were treated with the indicated drugs in the presence or absence of Chloroquine (1µM) or Baf-A1 (1nM) for 60 hr. Cell viability was assessed by MTT. Mean values ± SD for 3 repeats are shown. P-values were measured by t-test.

*p-value < 0.05, **p-value < 0.01, ***p-value < 0.001, ****p-value < $1x10^{-4}$

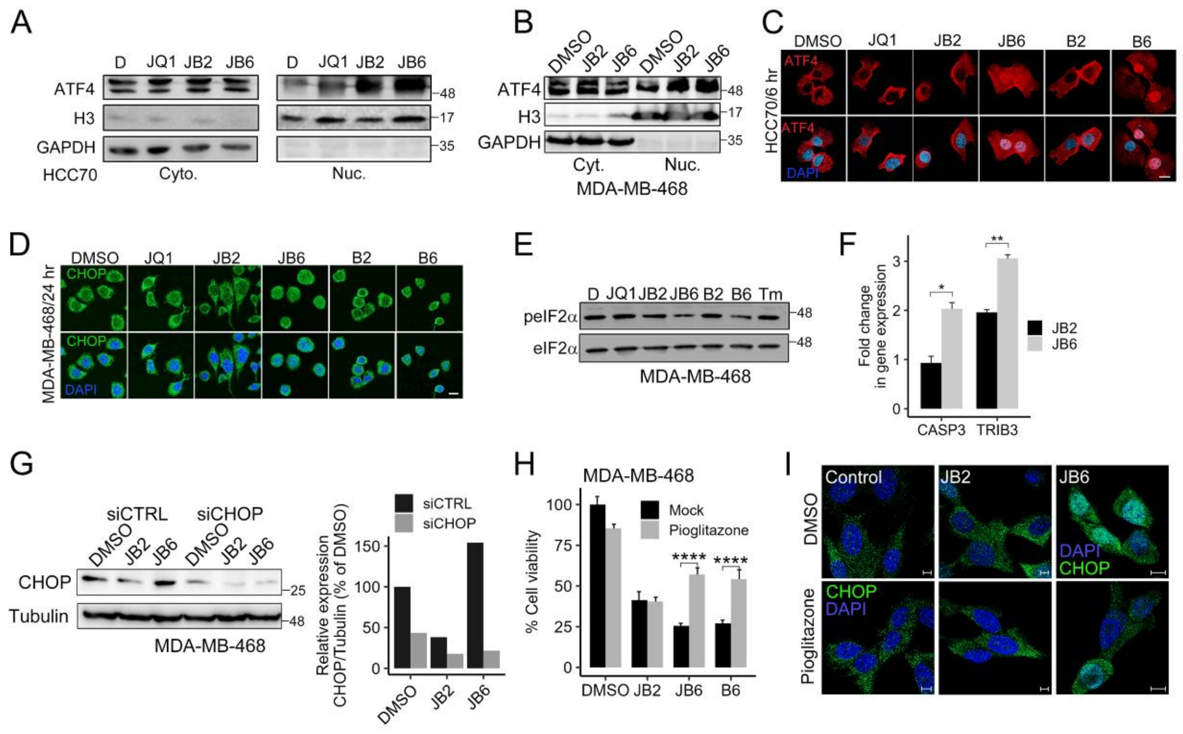

Figure S8: JB6-induced apoptosis is associated with ATF4/CHOP activation.

(A- C) ATF4 is enriched in the nuclear fraction of HCC70 (**A, C**) or MDA-MB-468 (**B**) cells at 6 hr (**C**) or 24 hr (**A, B**) post drugs treatment. Shown are representative WB (**A, B**) or IF (**C**) analysis. Scale bar, 10 μm. H3 (Histone 3) (nucleus) and GAPDH (cytoplasmic) were used as fractionation controls.

(D) CHOP is enriched in JB6 nucleus as shown by the confocal images of MDA-MB-468 in response to the indicated drugs treatments. Scale bar, 10 μm.

(E) Reduced phosphorylation of eIF2α was observed in B6- or JB6-treated MDA-MB-468 cells at 24 hr post treatment as shown by WB. Tunicamycin (Tm) 1 μg ml^{-1}.

(F) Transcription level of CHOP target genes *Casp3* and *Trib3* in response to the applied drugs for 24 hr was determined by qPCR. Data are present as fold of control (DMSO). Mean values of two independent experiments are shown. P-values were measured by t-test.

(G) MDA-MB-468 cells were transfected with siRNA CHOP or siCTRL and 48 hr later were treated with the indicated drugs for 24 hr. Left: Cell lysates were analysed by WB with the indicated antibodies. Right: densitometric analysis of CHOP bands shows siCHOP knock-down efficiency.

(H-I) Pioglitazone attenuated ER stress pathway and partially restored cell viability of JB6-treated MDA-MB-468 cells. Cells were treated with the indicated drugs and 72 hr later cell viability was measure by MTT. Shown are means ± SD of two independent experiments done in triplicates. P-values were measured by t-test (**H**). HCC70 cells were treated with JB2 or JB6 in the absence or presence of pioglitazone (10 μM) for 24 hr, and localization of CHOP was determined by IF. Representative confocal images are shown, scale bar 10μM (**I**).

*p-value < 0.05, **p-value < 0.01, ****p-value < $1x10^{-4}$

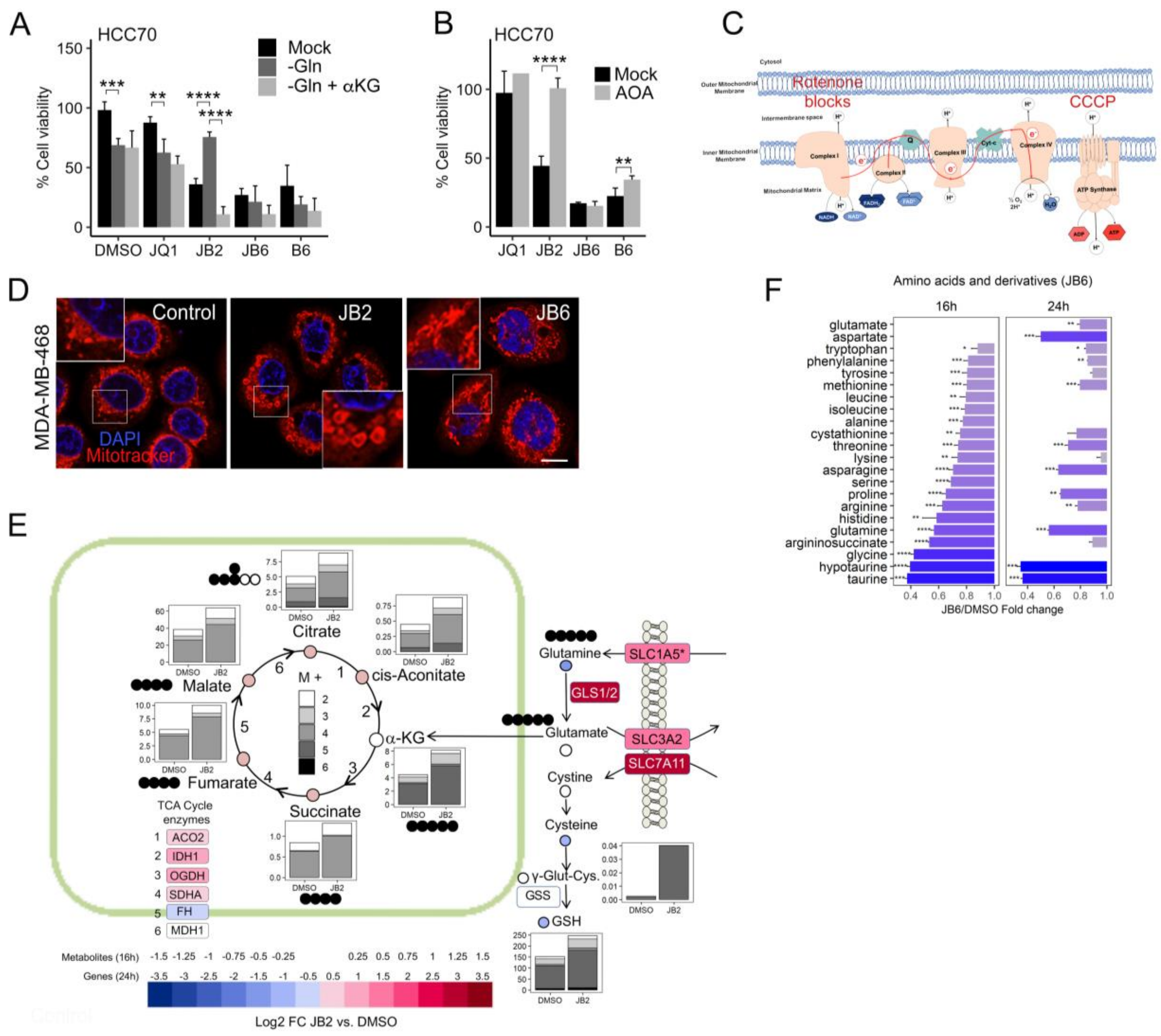

Figure S9: Glutaminolysis and mitochondria activity are essential for JB2 induced ferroptosis.

(A, B) HCC70 cells were treated with the indicated drugs in either regular (Mock) media, in glutamine free (-Gln) media, in glutamine free plus α-ketoglutarate (-Gln+ αKG) media (**A**), or in regular media plus AOA inhibitor (**B**). Cell viability was measured after 72 hr by MTT and was calculated relative to control (%). Mean values ± SD from two independent experiments done in duplicates. P-values measured by t-test.

(C) Rotenone and CCCP can block the electron transport chain.

(D) Mitochondrial morphology of MDA-MB-468 cells treated with the indicated drugs for 24 hr and stained with MitoTracker. Shown are representative confocal images. Scale bar, 5 µm.

(E) TCA cycle metabolites, genes and tracing results. Squares indicate genes, circles indicate the metabolites. The background color of the squares and circles indicate the fold change between JB2 and DMSO as detailed in the color bar below. Bar graphs shows the glutamine C^{13} tracing results (main isotopolouges) for these metabolites (colors depict carbon numbers (2-6), as shown in the legend in the center). The y- axis of the bar graphs are the normalized AUCs x10^6. Carbon tracing data are means of n = 5 (JB2) and n = 4 (DMSO) independent repeats.

(F) Polar metabolite screen was performed in JB6 and DMSO. Plots show the fold change in normalized intensities of metabolites (amino acids and derivatives) in JB6 vs. DMSO (3 individual repeats). P-values were determined by t-test, comparing JB6 to DMSO.

**p-value < 0.01, ***p-value < 0.001, ****p-value < $1x10^{-4}$
